# A novel signature model based on mitochondrial-related genes for predicting survival of colon adenocarcinoma

**DOI:** 10.1186/s12911-022-02020-3

**Published:** 2022-10-22

**Authors:** Hongli Gao, Fei Xing

**Affiliations:** 1 Department of Oncology, Shengjing Hospital of China Medical University, Shenyang, China; 2 Tumor Stem Cell and Transforming Medicine Laboratory, Shengjing Hospital of China Medical University, Shenyang, China

**Keywords:** Colon adenocarcinoma (COAD), Mitochondrial-related genes, Risk score, Overall survival (OS), Signature, Immune checkpoint

## Abstract

**Background:**

Colon cancer is the foremost reason of cancer-related mortality worldwide. Colon adenocarcinoma constitutes 90% of colon cancer, and most patients with colon adenocarcinoma (COAD) are identified until advanced stage. With the emergence of an increasing number of novel pathogenic mechanisms and treatments, the role of mitochondria in the development of cancer, has been studied and reported with increasing frequency.

**Methods:**

We systematically analyzed the effect of mitochondria-related genes in COAD utilizing RNA sequencing dataset from The Cancer Genome Atlas database and 1613 mitochondrial function-related genes from MitoMiner database. Our approach consisted of differentially expressed gene, gene set enrichment analysis, gene ontology terminology, Kyoto Encyclopedia of Genes and Genomes, independent prognostic analysis, univariate and multivariate analysis, Kaplan–Meier survival analysis, immune microenvironment correlation analysis, and Cox regression analysis.

**Results:**

Consequently, 8 genes were identified to construct 8 mitochondrial-related gene model by applying Cox regression analysis, CDC25C, KCNJ11, NOL3, P4HA1, QSOX2, Trap1, DNAJC28, and ATCAY. Meanwhile, we assessed the connection between this model and clinical parameters or immune microenvironment. Risk score was an independent predictor for COAD patients’ survival with an AUC of 0.687, 0.752 and 0.762 at 1-, 3- and 5-year in nomogram, respectively. The group with the highest risk score had the lowest survival rate and the worst clinical stages. Additionally, its predictive capacity was validated in GSE39582 cohort.

**Conclusion:**

In summary, we established a prognostic pattern of mitochondrial-related genes, which can predict overall survival in COAD, which may enable a more optimized approach for the clinical treatment and scientific study of COAD. This gene signature model has the potential to improve prognosis and treatment for COAD patients in the future, and to be widely implemented in clinical settings. The utilization of this mitochondrial-related gene signature model may be benefit in the treatments and medical decision-making of COAD.

**Supplementary Information:**

The online version contains supplementary material available at 10.1186/s12911-022-02020-3.

## Introduction

According to the 2018 Global Cancer Data Report, colorectal cancer (CRC), including colon adenocarcinoma (COAD), is now among the top three cancers in terms of morbidity and ranks second with respect to mortality [1, 2]. Currently, the prognosis for COAD is largely determined by clinicopathological characteristics and the stage of the tumor [3, 4]. The primary COAD treatments include surgery, radiotherapy, and chemotherapy. 5-Fluorouracil (5-FU) and folinic acid (leucovorin), which are combined with oxaliplatin (FOLFOX) or irinotecan, represent one of the highest standards among these therapies (FOLFIRI). Although effective early screening improved recurrence, and additional treatment options have contributed to a decline in the incidence and mortality of COAD, many patients continue to be diagnosed at an advanced stage. In recent years, the average age of onset has decreased, and the 5-year survival rate of patients with distant metastases is under 10% [5]. Since early symptoms of COAD are not readily apparent, most patients have already entered advanced stage when diagnosed. Over 50% of COAD patients are diagnosed in their advanced stages [5]. For the development of effective treatment strategies, it is pivotal to conduct additional investigation on carcinogenesis of COAD to probe new and promising biomarkers. During tumor cell formation, the metabolism is reprogrammed to rapidly facilitate cancer cell growth. The central role of mitochondria in this process is critical. Pan-cancer mitochondrial-gene analysis displayed that mitochondrion genomic alterations and nuclear mitochondrial were closely associated with 38 tumor types [6]. Some researches had showed the roles of mitochondrial genes and its relationship with the survival status of cancer patients. But the efficacy of mitochondrial-related genes in evaluating COAD patients’ prognosis lacks depth researches [7]. Thus, our research aims to investigate whether transcriptomic profiling of mitochondrial genes is connected to the prognosis of COAD patients.

Mitochondria are unique organelles that carry extranuclear genetic material, and they are associated with a variety of metabolic diseases, degenerative diseases, age-related human diseases, and cancer [8, 9]. Mitochondria is evidenced to exert a significant part in the carcinogenesis and progression of COAD through retrograde regulation of the nucleus [10]. Furthermore, reactive oxygen species (ROS) produced in mitochondria can promote the proliferation and migration of tumor cells [11]. Accordingly, research into mitochondria is extensively acknowledged in a variety of fields. More recently, it has been demonstrated that mitochondria from non-tumor cell lines inhibit tumor formation in the same nuclear context, including inhibition of apoptosis, proliferation, anoxic survival, drug resistance, colony formation, and invasion, as well as enhanced tumor cell response to therapy [12]. In addition, the bidirectional communication between mitochondria and the nucleus facilitates retrograde regulation of the nucleus [13]. During the formation of tumor cells, metabolism is reprogrammed to facilitate rapid proliferation of cancer cells. Mitochondria play an indispensably pivotal role in this process. Mitochondrial gene analysis of pan-cancer revealed that nuclear mitochondrial genomic alterations were closely associated among 38 tumor types [6]. There are many studies to explore the functions of mitochondrial-related genes in cancer and how they are connected to prognosis. However, research on the role and effectiveness of mitochondrial-related genes in predicting the prognosis of COAD is insufficient [7]. Mitochondria could become innovative target for anti-cancer drugs, and the role of mitochondria-related genes in cancer prognosis prediction may become a novel and potential diagnostic model. Therefore, the object of this research is to excavate whether transcriptomic profiling of mitochondrial genes correlates with the prognosis and survival of COAD patients.

Recent evidence indicates that the combination of microarray technology and bioinformatics tools can effectively identify new targets concerning diagnosis, and prognosis of cancer [14, 15]. Therefore, bioinformatics is a feasible tool for filtering DEG and questing key genes [11, 12]. Using RNA sequencing data from TCGA database in conjunction with bioinformatics and statistical methods, we aimed to systematically discuss the effect of mitochondria-related genes in COAD. Initially, we analyzed 1613 mitochondrial-related genes and corresponding clinical data from TCGA of COAD patients. Then, we identified 249 mitochondrial-related genes with differential expression in COAD patients. Secondly, we developed a novel 8-gene signature prognosis model by employing Cox regression analysis to screen 8 significant genes that influence the survival of COAD patients. Thirdly, we validated the prognostic efficacy of the established mitochondrial-related gene pattern utilizing validation dataset GSE39582. On the basis of this signature model, a nomogram and good AUC curves were developed to demonstrate the predictability and stability of the model. Finally, the functional signaling pathways, immune checkpoints and immune cell fraction in the tumor microenvironment, and clinical parameters between high- and low-risk groups were further investigated and analyzed. The novel 8 mitochondria-related gene pattern to assess prognosis in COAD provided essential bioinformatics evidence to advance understanding of the complex mechanisms of the COAD progression and to optimize prognosis and improve survival of COAD patients.

## Methods

### Data origin and collection

RNA-seq transcriptome data of 480 COAD and 41 normal samples, along with corresponding clinical parameters of COAD patients, were downloaded from TCGA-COAD cohort (https://portal.gdc.cancer.gov/). GEO dataset GSE39582 (https://www.ncbi.nlm.nih.gov/geo/) comprising 566 colorectal cancer patients was defined as an independent validation dataset. Patients without survival information were excluded. Mitochondrion-related genes were downloaded from the MitoMiner database [16], which collected human genes encoding proteins associating with mitochondria and affecting their form and function. The most recent update to MitoMiner is version 4.0, which includes 1613 mitochondrial-related genes.

### DEGs analysis

Differential expression of mitochondrion-related genes was analyzed by “limma” package (R v3.6) [17] with the cut-off *P* < 0.05 and abs(logFC) > 1. The expression of mitochondrion-related DEGs was compared between tumor and normal tissue utilizing heatmaps and volcano diagrams.

### Functional enrichment analysis

Using the JAVA program gsea-3.0.jar, the GSEA was carried out on the gene ontology gene set of MSigDB to illustrate differences between normal tissue samples and COAD samples [18–20]. The algorithm of random sampling consisted of 1,000 permutations. Employing a false discovery rate (FDR) < 0.05, an enrichment between two types was identified. The "clusterProfiler" [21], "ggplot2" [22], and "GOplot" R packages [23] were utilized to perform GO and KEGG analyses n tumor tissue versus normal tissue.

### Model construction based on differential mitochondrial-related genes

To unearth mitochondrion-related DEGs value, a univariate Cox analysis of OS was performed. We performed boxplot diagrams to visualize the expression of prognostic-related genes. The multivariate Cox analysis was leveraged to establish a prognostic pattern to minimize the hazard of overfitting. Normalized expression of each gene and their regression coefficients were utilized to compute risk scores. The formula was as follows: score = ESUM (expression of each gene × homologous coefficient). Riskscore = 0.53*CDC25C + 0.64*NOL3 + 0.601*QSOX2 + 0.281*KCNJ112.44*DNAJC28 + 1.294*ATCAY-0.604*TRAP1 + 0.436*P4HA1. Patients were stratified into high-risk and low-risk groups based on median risk score. For survival analysis, the optimal cut-off expression value was resolved by the "surv cutpoint" of "survminer" R package [24]. Based on expression of each prognostic-related gene, Kaplan–Meier curves were utilized to juxtapose OS between two subgroups. To compare the correlation between clinicopathological variables and risk score, univariate and multivariate Cox analyses were performed. Independent cohort GSE39582 was retrieved to validate the model.

### Construction of nomogram, ROC curves clinical features, and immune status for COAD

The "survivalROC" R package [25] was used to assess predictive worth of the gene pattern using time-dependent receiver operating characteristic (ROC) curve analyses. RMS package [26] was utilized to generate nomograms that incorporated clinically significant characteristics and risk scores. The relationship between clinicopathological variables and risk score was assessed by student's t-test. Visual data representations were produced using R package “beeswarm” [27]. The correlation of immune checkpoints and immune cell infiltration fraction with risk score was also calculated by spearman correlation.

### Statistical analysis

All statistical analyses were conducted by R package (v. 3.6.3). The Kaplan–Meier analysis with log-rank test was used to determine the significance of the difference in survival rates among risk groups. P values were adjusted utilizing Benjamini–Hochberg method. *P* < 0.05 was regarded significant.

## Results

### Flow chart of overall design

**Fig. 1:**
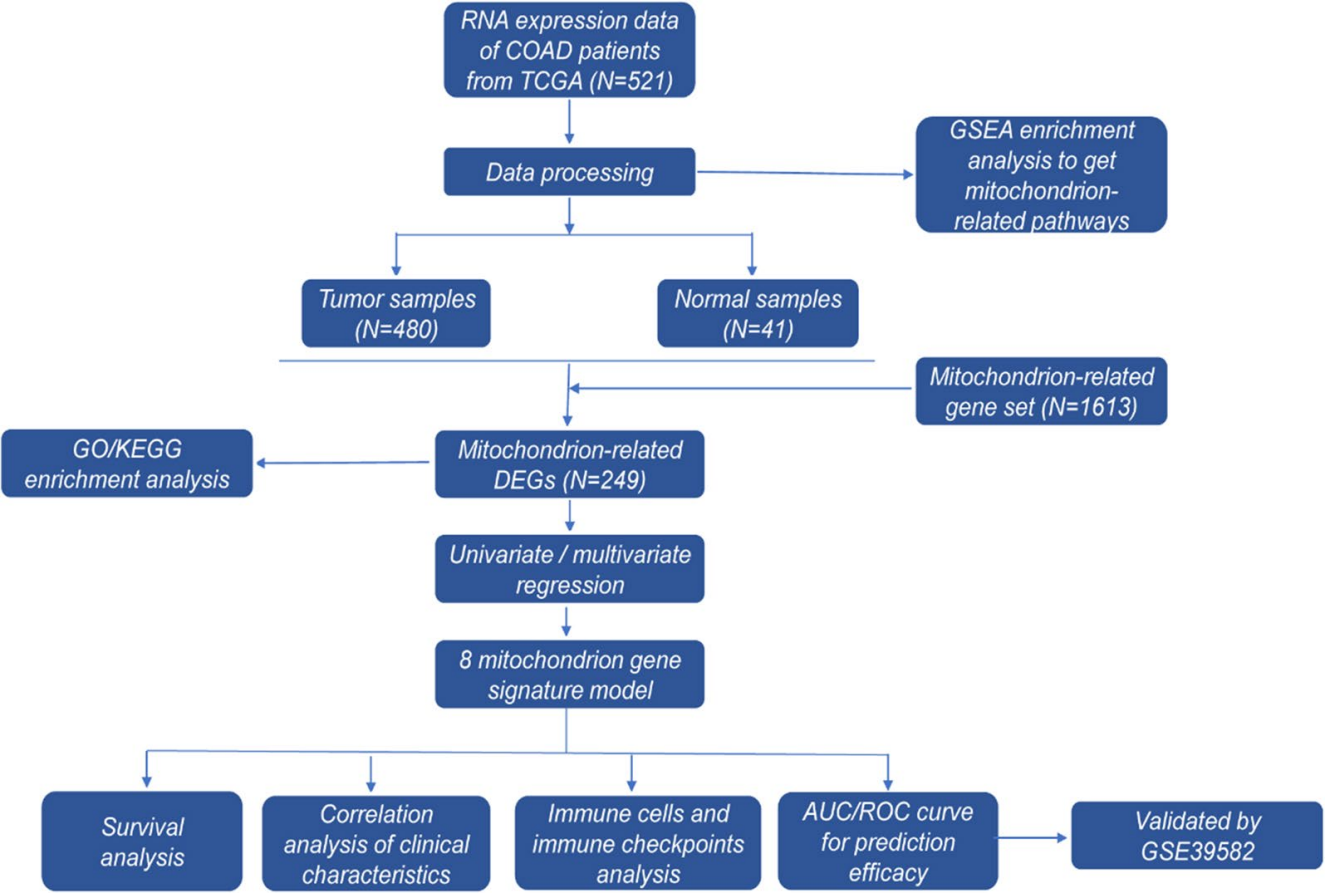
The flow diagram for the establishment of the mitochondrial-related gene signature model

521 COAD patients from the TCGA-COAD cohort were enrolled totally, including both tumor (n = 480) and normal samples (n = 41) (Fig. 1A). Upon downloading the RNA expression data for COAD patients from TCGA, the GSEA enrichment analysis was used to identify various mitochondrial-related pathways. These pathways enriched in mitochondrion-related metabolism prompted us to investigate the connection between mitochondrial metabolism and COAD pathogenesis further. We obtained the mitochondrion-related gene set (n = 1613) from MitoMiner database in a previous study by Anthony C Smith. The mitochondrion-related gene set was intersected with DEGs from TCGA-COAD datasets to obtain "differentially expressed mitochondrion-related genes" (n = 249). Next, we analyzed each gene using univariate/multivariate Cox regression, ultimately selecting eight genes for establishing the signature model. By using, univariate/multivariate Cox regression, correlation analysis, and Kaplan–Meier analysis, the relationship among the 8 gene signature model, clinical characteristics, immune checkpoint, and significance of survival were explored further. Finally, the nomogram graph and area under (AUC)/ receiver operating characteristic (ROC) curve were constructed to validate the efficacy. GSE39582 cohort also was applied to validate accuracy of the signature model.

### Identification of differential metabolic gene sets between COAD tumors and normal tissue

**Fig. 2:**
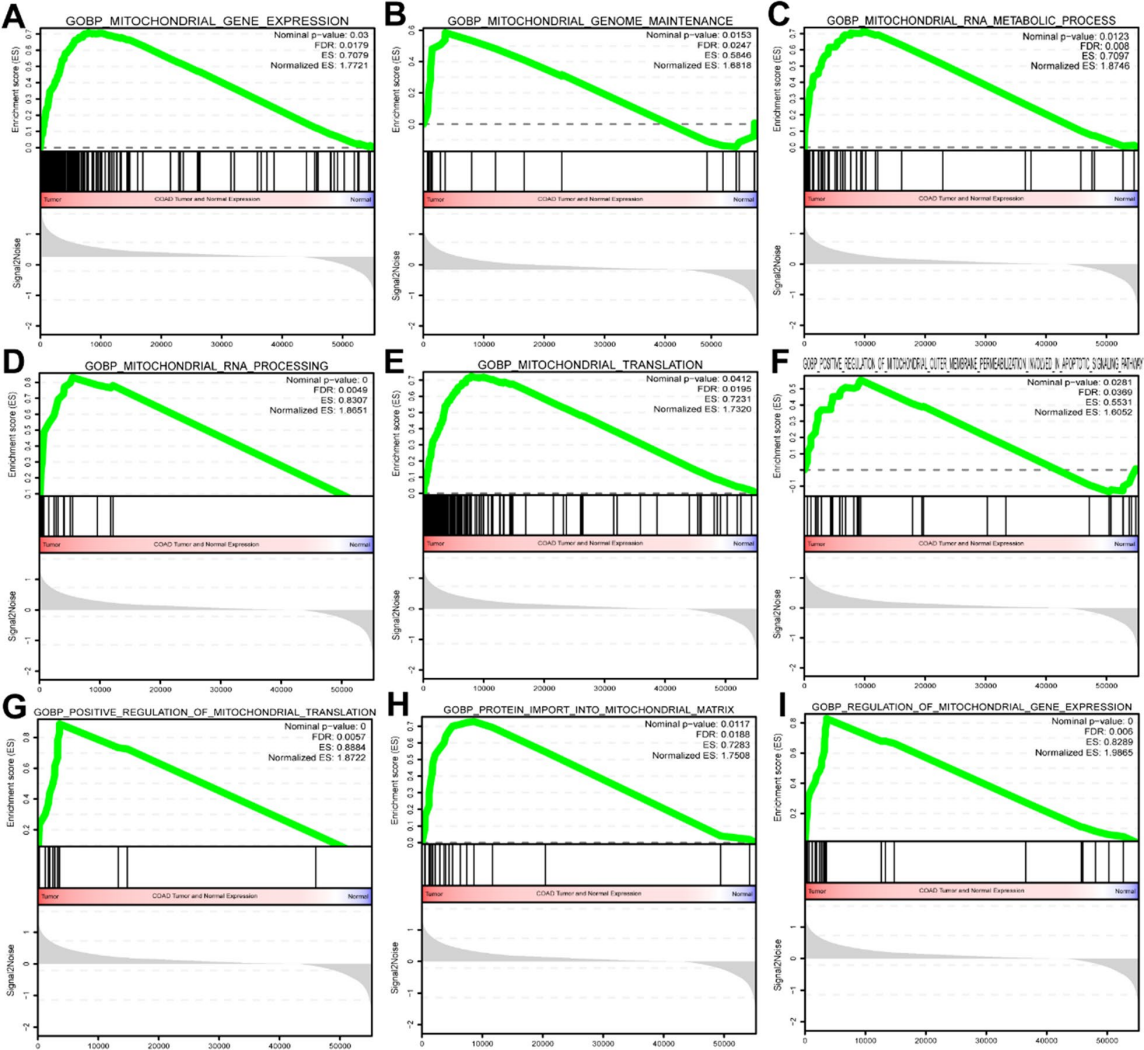
Gene set enrichment analysis of mitochondrion-related pathways of tumor in TCGA-COAD. The enrichment items were selected with a normalized *P* value 0.05. NES: normalized enrichment score; FDR: false correction rate

Although it has been reported that COAD tumorigenesis exhibits a unique relationship with mitochondrial metabolic processes, the associated metabolic significance remains unknown. GSEA evidenced that in nine associated metabolic pathways (NOM *P* < 0.05) (Fig. 2), the gene sets were significantly enriched. These pathways included mitochondrial gene expression (NES = 1.7721, NOM *P* = 0.03), mitochondrial genome maintenance (NES = 1.6818, NOM *P* = 0.0153), mitochondrial RNA metabolism (NES = 1.8746, NOM *P* = 0.0123), mitochondrial RNA processing (NES = 1.8651, NOM *P* < 0.001), mitochondrial translation (NES = 1.7320, NOM *P* = 0.0412), positive regulation of mitochondrial translation (NES = 1.8722, NOM *P* < 0.001), positive regulation of mitochondrial outer membrane permeabilization involved in apoptotic signaling pathway (NES = 1.6025, NOM *P* = 0.0281), protein import into mitochondrial matrix (NES = 1.7508, NOM P = 0.0117), and regulation of mitochondrial gene expression (NES = 1.9865, NOM *P* < 0.001) (Table 1).

**Table 1:** 9 mitochondrion-related GSEA pathways

NAME	SIZE	ES	NES	NOM p-val	FOR q-val	FWER p-val	RANKAT MAX	LEADING EDGE
GOBP_REGULATION_OF_MITOCHONDRIAL_GENE_EXPRESSION	28	0.828871	2.004785	0.002037	0.004225	0.175	3584	tags = 68%, list = 6%, signal = 73%
GOBP_MITOCHONDRIAL_RNA_PROCESSING	16	0.830658	1.874972	0	0.012356	0.454	5467	tags = 81%, list = 10%, signal = 90%
GOBP_POSITIVE_REGULATION_OF_MITOCHONDRIAL_TRANSLATION	16	0.888397	1.870809	0	0.012631	0.463	3584	tags = 81%, list = 6%, signal = 87%
GOBP_MITOCHONDRIAL_RNA_METABOLIC_PROCESS	44	0.709743	1.833979	0.014463	0.016309	0.542	10,154	tags = 75%, list = 18%, signal = 92%
GOBP_MITOCHONDRIAL_GENE_EXPRESSION	161	0.707892	1.757023	0.029724	0.028204	0.703	7928	tags = 65%, list = 14%, signal = 76%
GOBP_PROTEIN_IMPORT_INTO_MITOCHONDRIAL_MATRIX	20	0.728259	1.742189	0.012048	0.030894	0.723	8594	tags = 80%, list = 16%, signal = 95%
GOBP_MITOCHONDRIAL_TRANSLATION	132	0.72311	1.716128	0.044304	0.036107	0.757	7928	tags = 67%, list = 14%, signal = 79%
GOBP_MITOCHONDRIAL_GENOME_MAINTENANCE	21	0.58461	1.634145	0.023033	0.056558	0.872	3623	tags = 48%, list = 7%, signal = 51%
GOBP_POSITIVE_REGULATION_OF_MITOCHONDRIAL_OUTER_MEMBRANE_PERIMEABI_LIZATION_INVOLVED_IN_APOPTOTIC_SIGNALING_PATHWAY	35	0.553105	1.611109	0.014286	0.063709	0.899	9343	tags = 63%, list = 17%, signal = 76%

### Functional analyses of differentially expressed mitochondrion-related genes in TCGA

**Fig. 3:**
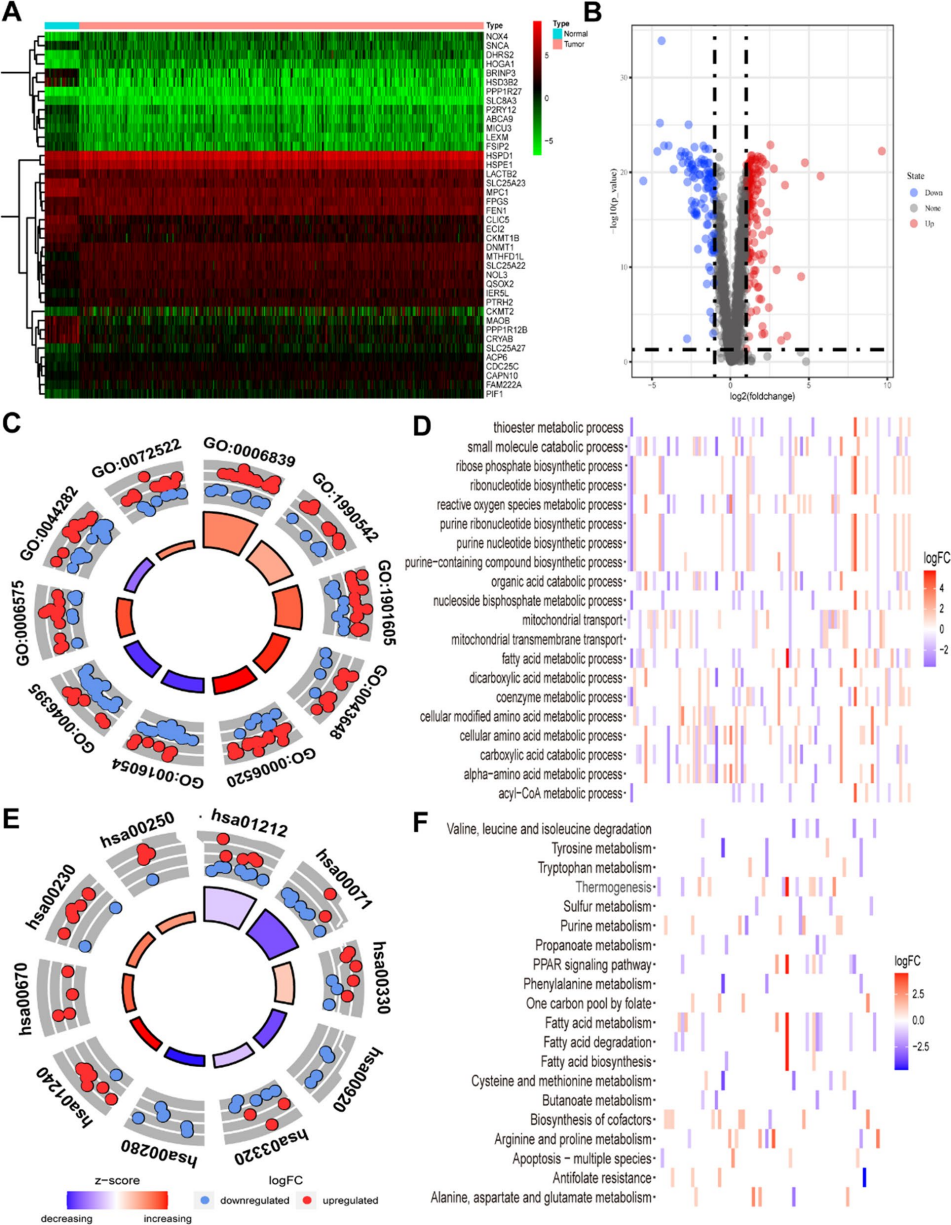
Functional analyses of differentially expressed mitochondrion-related genes in TCGA. **A** The heat map of mitochondrion-related DEGs in TCGA-COAD. Red: high expression level. Green: low expression level. **B** The volcano map of mitochondrion-related DEGs in TCGA-COAD. Red: upregulated genes. Blue: downregulated genes. Gray: no-significant difference genes. **C**–**D** The most significant GO and KEGG enrichment pathways in the TCGA cohort in up-regulated genes group. **E**–**F**: The most significant GO and KEGG enrichment pathways in the TCGA cohort in up-regulated genes group. Respectively, the enriched items were filtered with a corrected *P* value 0.05; the length and color of the point represent the absolute value of NES and the *q* values.

We further investigated the relationship between COAD and mitochondrial metabolism as GSEA revealed the gene sets were significantly enriched in nine associated metabolic pathways. We identified 249 mitochondrion-related DEGs by intersecting the mitochondrion-related gene set with the TCGA-COAD DEG datasets (Additional file 3: Table S1). These mitochondrial-related DEGs appeared on volcano and heat maps (Fig. 3A–B). To clarify the biological implication connected with these mitochondrial-related DEGs, GO and KEGG analyses were performed on DEGs. Expectedly, DEGs were enriched in mitochondrial metabolism, including transport processes and fatty acid metabolism (*P* < 0.05) (Fig. 3C–F). Moreover, the DEGs were considerably enriched in a number of other biological processes, such as thermogenesis, the peroxisome proliferator-activated receptor (PPAR) signaling pathway, and apoptosis in multiple species.

### The establishment of a mitochondrion-related prognostic model

**Fig. 4:**
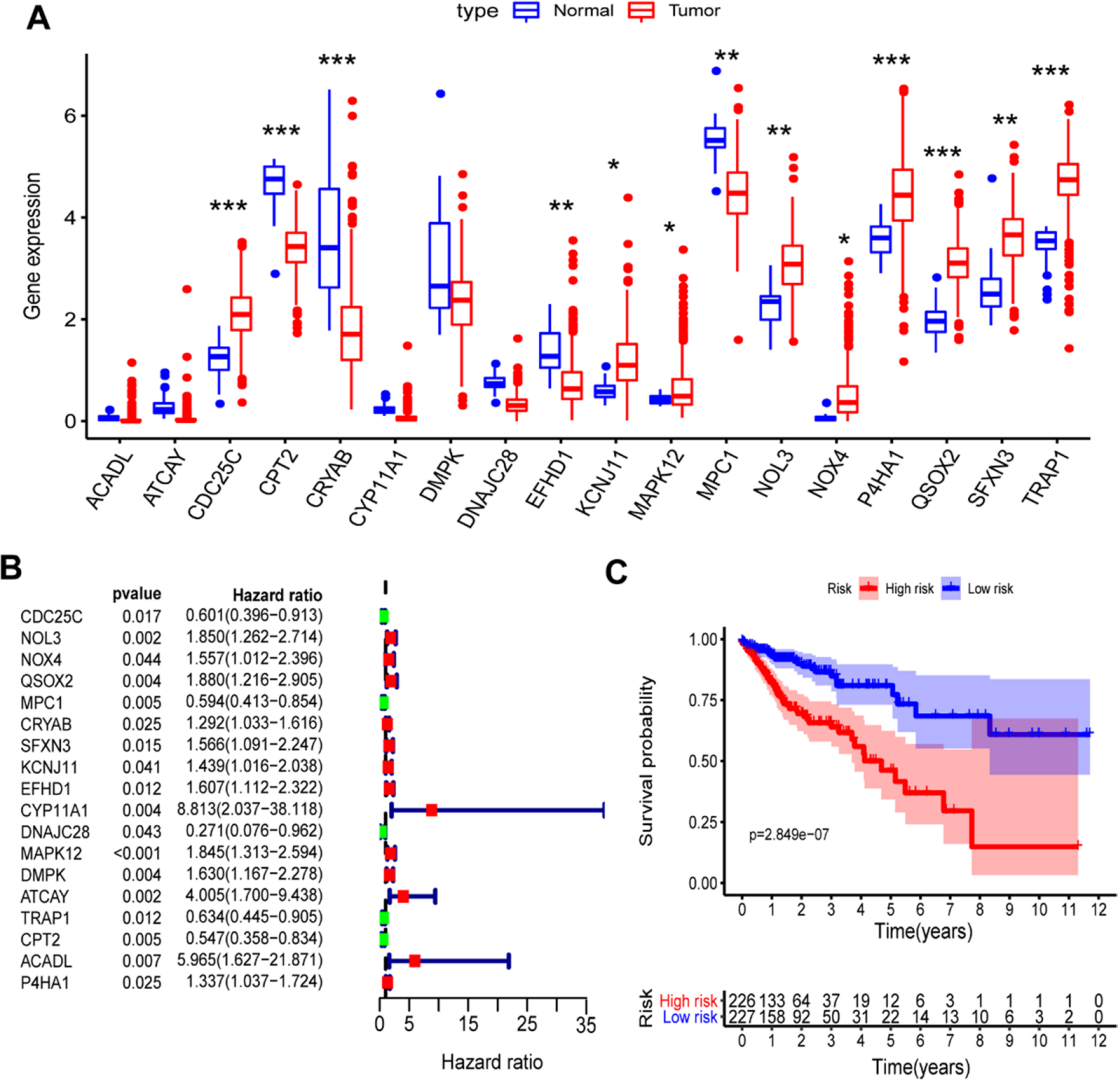
Predictive model construction according to Cox regression analysis. **A** The expression of 18 independent prognostic factors between tumor and normal tissue in TCGA-COAD. **B** The forest plot of the relation between the expression levels of 18 genes and OS in TCGA-COAD patients. Hazard ratios (HR), *P* value, and corresponding 95% confidence intervals were evaluated by univariate Cox regression analyses. **C** Kaplan–Meier OS curves for patients with high- and low-risk score group in the TCGA-COAD cohort

**Fig. 5:**
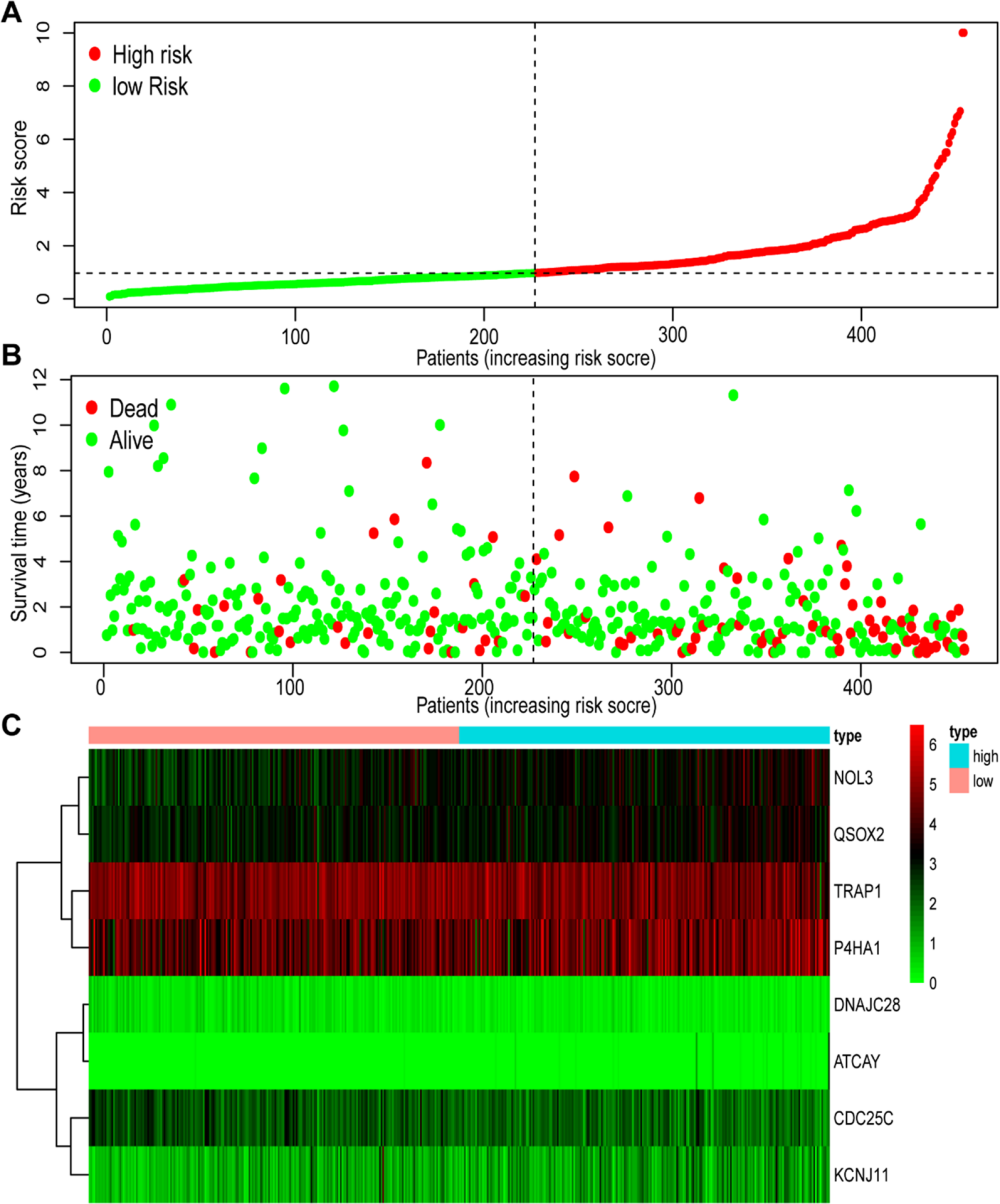
Prognostic values of the eight-gene signature model in TCGA-COAD cohort. **A** The distribution and median value of the risk scores between high- and low- risk score group in the TCGA-COAD cohort. **B** The risk distribution curve of OS status, OS and risk score between high- and low- risk score group in TCGA-COAD cohort. **C** The heat map of eight-genes expression level between high- and low- risk score group in TCGA-COAD cohort. Red: high expression level. Green: low expression level

To identify genes significantly associated with prognosis, a univariate Cox analysis was applied. 18 mitochondrion-related were initially identified as prognostic genes (Fig. 4A–B). Figure 4A displays18 mitochondrion-related differential genes in COAD and normal tissues. Figure 4B depicts the results of the univariate Cox analysis. Next, a prognostic pattern according to multivariate Cox analysis was developed (Additional file 4: Table S2). As displayed in Fig. 4C and 5A, a risk score was computed, as detailed in Materials and Methods section. A high-risk group and a low-risk group were divided according to median risk score (*P* < 0.001). High-risk patients often die earlier than low-risk patients (Fig. 5B). As for this scatter plot, every point just represents a patient. Tumors are heterogeneous, and each clinical patient is also specific. Analyzing this issue from a clinical point of view, patients assigned to a high-risk group do not absolutely have a worse prognosis, whereas patients assigned to a low-risk group do not necessarily have a longer survival time. Our model efficacy is decided by the final proportions and probability.

**Fig. 6:**
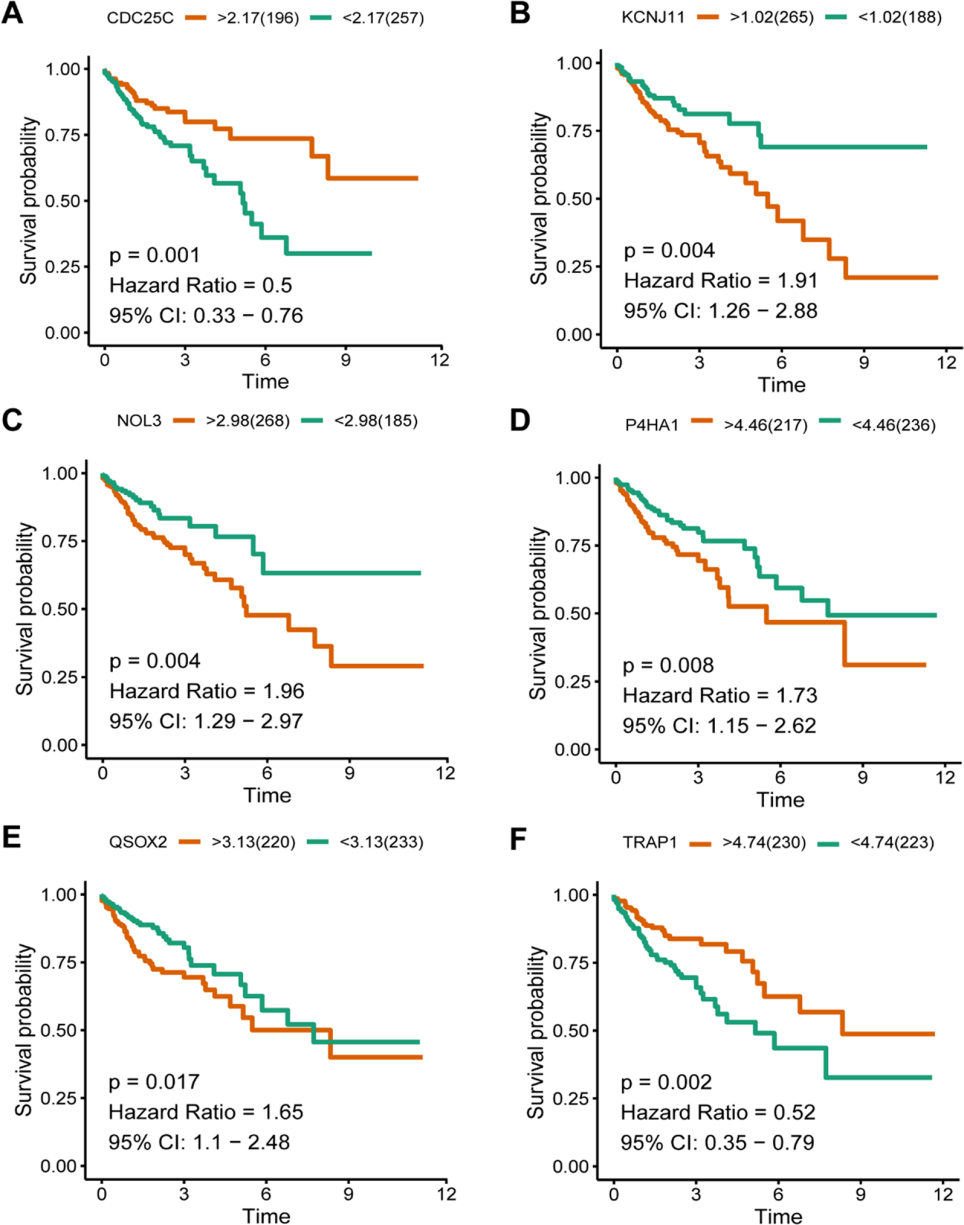
Kaplan–Meier plots of the prognostic mitochondrion-related gene signatures. **A**–**F**: The six genes included CDC25C, KCNJ11, NOL3, P4HA1, QSOX2 and TRAP2 (*P* < 0.05). Another two genes were displayed in Additional file 1: Fig. S2 due to their *P* value > 0.05

Eight genes were involved in the mitochondrion-related pattern. Kaplan–Meier plots revealed that eight genes were identified as independent prognostic signatures (*P* < 0.05) (Fig. 6A–F), involving CDC25C [*P* = 0.001, Hazard Ratio (HR) = 0.5], KCNJ11 (*P* = 0.004, HR = 1.91), NOL3 (*P* = 0.004, HR = 1.96), P4HA1 (*P* = 0.008, HR = 1.73), QSOX2 (*P* = 0.017, HR = 1.65), and TRAP1 (*P* = 0.002, HR = 0.52), and two genes with *P* value > 0.05, DNAJC28 (*P* = 0.051, HR = 0.66) and ATCAY (*P* = 0.206, HR = 1.3) (Additional file 1: Fig. S1), involving DNAJC28 (*P* = 0.051, HR = 0.66) and ATCAY (*P* = 0.206, HR = 1.3). A heat map depicts the expression of eight mitochondrion-related signatures in COAD (Fig. 5C).

**Fig. 7:**
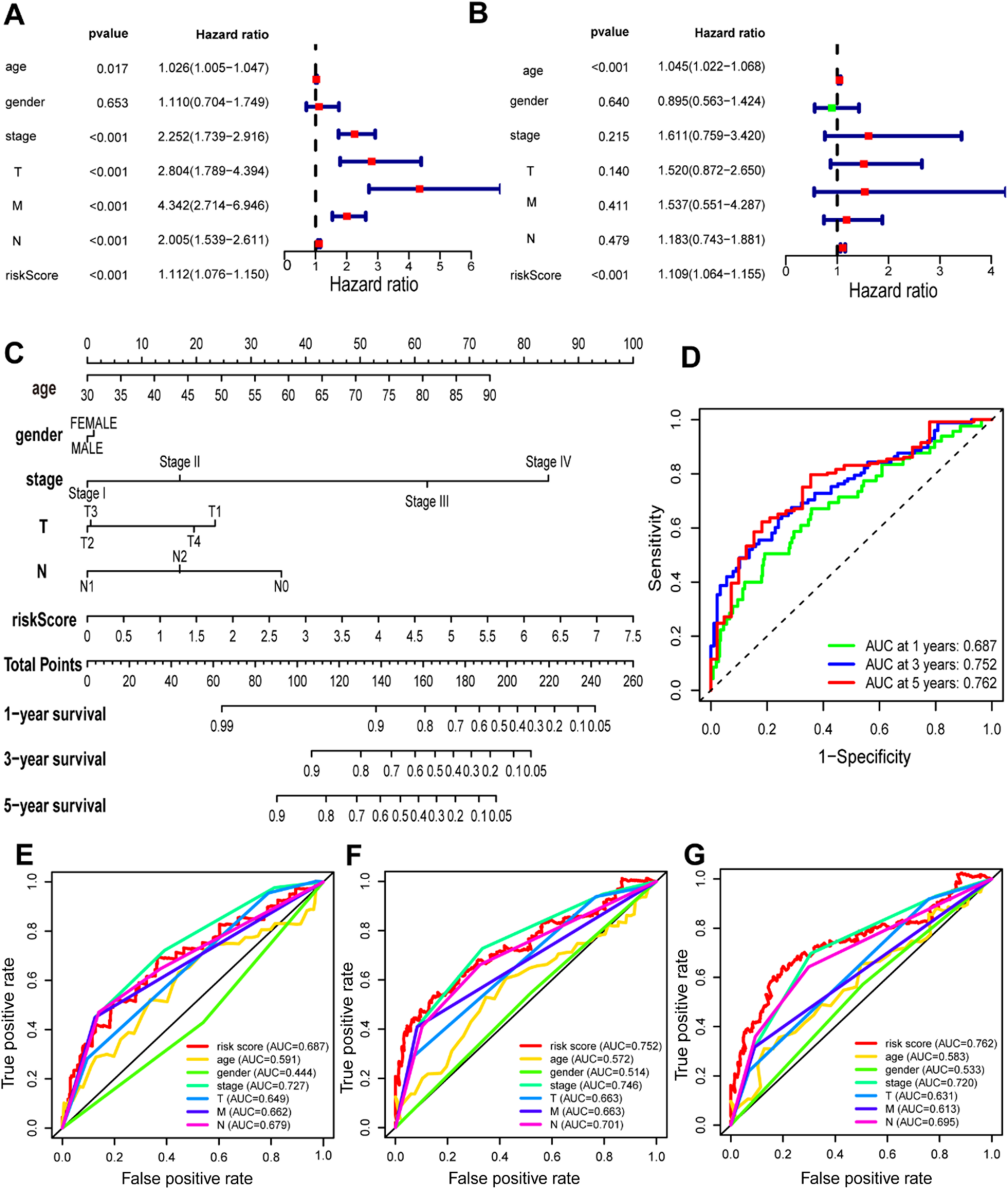
Univariate and multivariate Cox regression, nomogram, and ROC curve regarding OS in the TCGA cohort. **A** Univariate Cox regression analyses regarding OS in the TCGA cohort. **B** Multivariate Cox regression analyses regarding OS in the TCGA cohort. **C** Nomogram for predicting OS probabilities in 1-, 3- and 5-year of TCGA-COAD patients. The point scale was used to arrange points to these variables. The sum of points arranged to each variable was rescaled to a range from 1 to 100. The points of the variables were accumulated and recorded as the total scores. The 1-, 3-, and 5-year survival probabilities of COAD patients were determined by drawing a vertical line directly from the total score axis down to the outcome axis. **D** The ROC curve for predicting 1-, 3- and 5-year OS of the nomogram in the TCGA-COAD cohort. **E**–**G** The ROC curve for predicting 1-, 3- and 5-year OS of the nomogram compared between this risk score model and other clinical characteristics in the TCGA-COAD cohort, including age, gender, stage, T stage, M stage and N stage

Equipped with the available variables, univariate and multiple Cox analyses were performed to clarify whether risk score was independent for OS. In univariate Cox analyses, the risk score was strongly associated to OS in TCGA-COAD cohort (*P* < 0.001, HR = 1.112) (Fig. 7A). These included variables are important parameters used in the clinical treatment of colon adenocarcinoma to measure its disease progression, grading and staging. Age and gender can determine the pathogenic factors of the patient, while T, N, M, stage can indicate the severity of the disease. By comparing the risk score with these typical variables, we can show that it has a relatively better predictive value for prognosis. In a multivariate Cox analysis, after adjusting for other confounding factors, the risk score remained independent for OS (*P* < 0.001, HR = 1.109) (Fig. 7B). After multiple Cox analysis combined with clinical stage and risk score and the development of a prognostic prediction pattern, the nomogram was conducted. This was used to confirm the model’s risk score as a prognostic factor to assess the predicted probability of OS at 1-, 3- and 5- years (Fig. 7C). It has been demonstrated that the model is effective in predicting OS at 1-, 3-, and 5- years (Risk score-AUC: 1-year -: 0.687, 3-year: 0.752, 5-year: 0.762) (Fig. 7D). In addition, we conformed risk score and clinical representative characteristics into the same ROC curve to compare their 1-, 3-, and 5-year prediction efficacy (Fig. 7E–G).

**Fig. 8:**
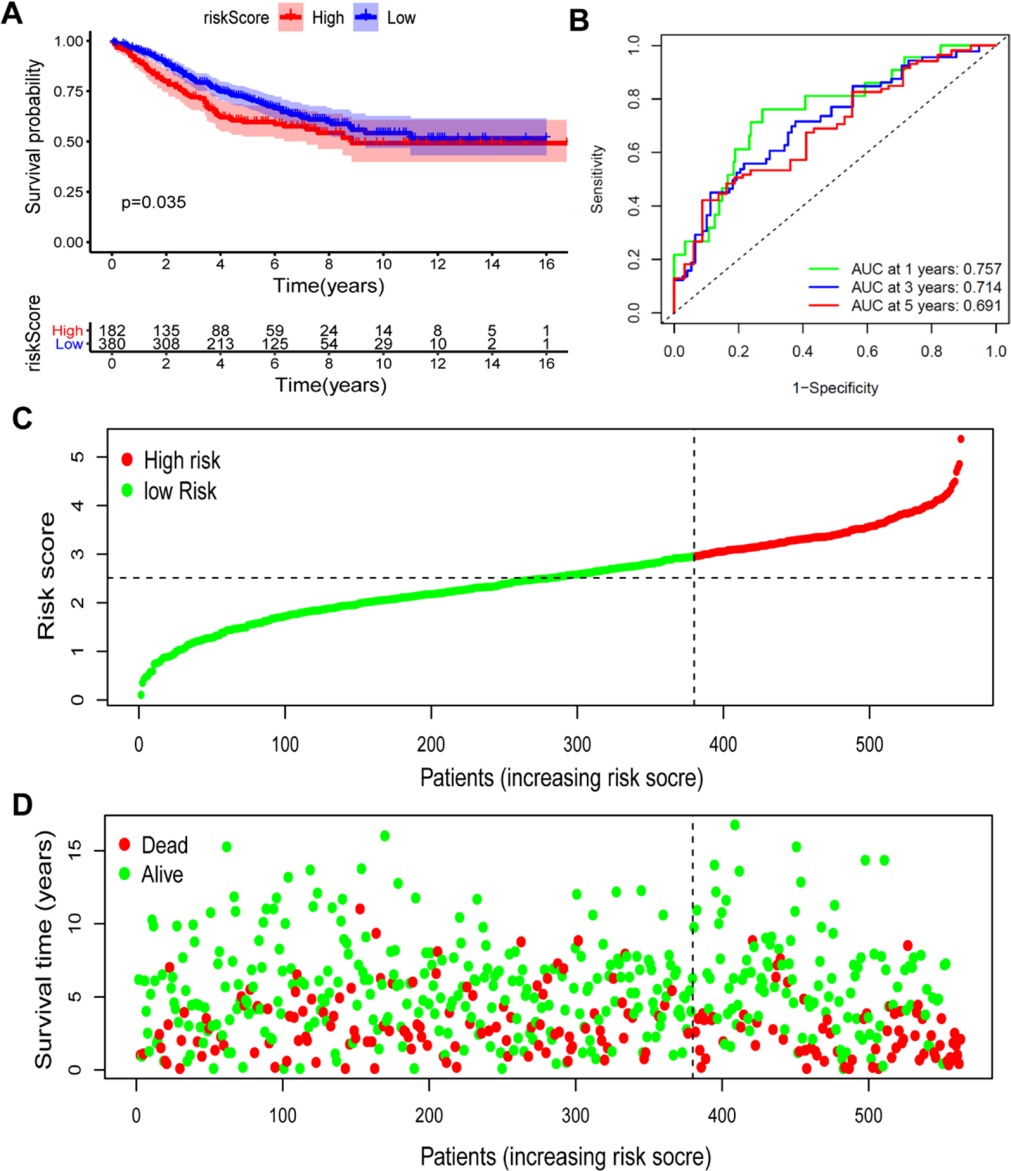
The validation of prognostic value for the mitochondrial-related gene signature model in GSE39582 cohort. **A**: Kaplan–Meier OS curves for patients with high- and low-risk score group in GSE39582 cohort. **B**: The ROC curve for predicting 1-, 3- and 5-year OS in GSE39582 cohort. **C** The distribution and median value of the risk scores in GSE39582 cohort. **D** The risk distribution curve of OS status, OS and risk score in TCGA-COAD cohort

Additionally, the prediction efficacy was validated in GSE39582 (Fig. 8A–C). The ROC curve was also validated in GSE39582 dataset, and 1-year risk score-AUC value: 0.757, 3-year risk score-AUC value: 0.714, 5-year risk score-AUC value: 0.691 (Fig. 8D). With respect to these data, Additional file 1: Fig. S2 displays the Decision Curve Analysis for this risk score. We developed two models; both of which included risk score and excluded risk score. It is evident that the risk score model appears to offer more advantages.

### Connection between risk score and clinicopathological characteristics

**Fig. 9:**
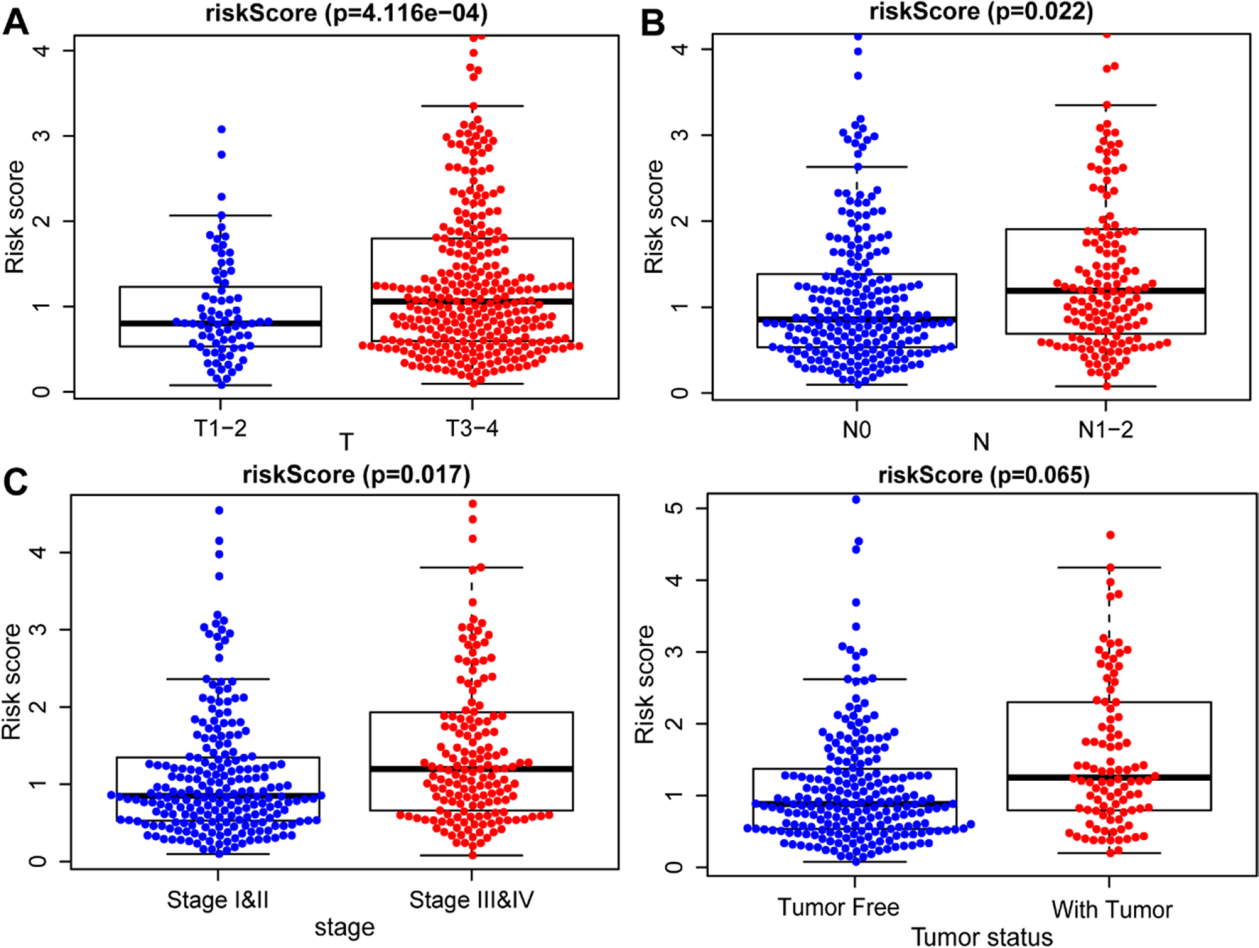
Association between risk score and clinicopathological characteristics (*P* < 0.05). **A**–**D** The statistical analyses between T, N, stage, and tumor status between high- and low-risk score group

Utilizing clinical information from the TCGA-COAD cohort, the current research probed the connection between risk score and prognostic factors. Results revealed a significant connection between higher risk scores and higher tumor (*P* = 4.116e-04), node (*P* = 0.022), and stage (*P* = 0.017) levels, as well as with tumors (*P* = 0.065) (Fig. 9). Other important clinical characteristics were not significantly interrelated with gender (*P* = 0.360), M stage (*P* = 0.107), histological type (*P* = 0.613), carcinoembryonic antigen (CEA) level (*P* = 0.313), lymphatic (P = 0.658) or perineural invasion (*P* = 0.450), which have been each reported to be correlated with COAD prognosis (Fig. S3).

### Connection between risk score and immune status in tumor microenvironment

**Fig. 10:**
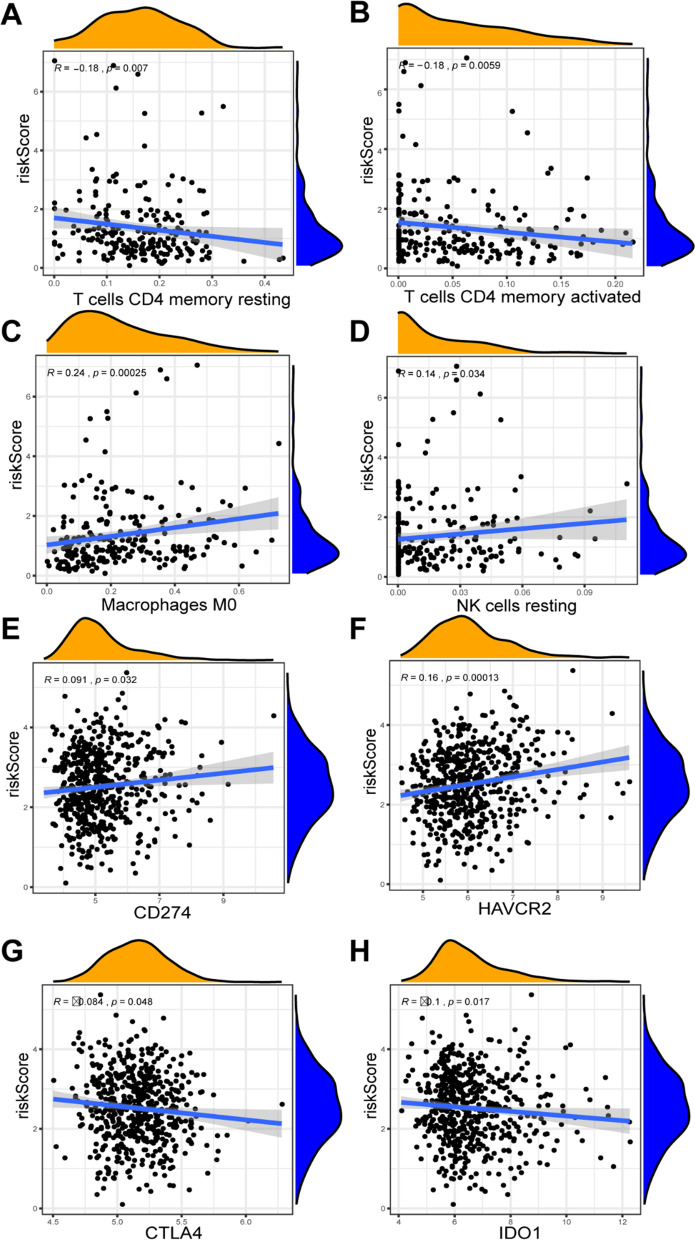
Correlation analysis between risk score and immunological status. **A**–**D** The correlation between risk score and immune cell infiltration, including CD4 memory resting T cells, CD4 memory activated T cells, M0 macrophages and resting NK cells. **E**–**H** The correlation between risk score and immune checkpoint expression, including CD274, HAVCR2, CTLA4 and IDO1

Immunotherapy is an emerging treatment for COAD. Increasingly related target mechanism research and clinical trials are in progress. Immunotherapies that inhibit immune checkpoints and target specific immune cell are common and effective immunotherapies in clinical practice. To discuss the fesible connection between our risk score model and immune cell infiltration and immune checkpoints, we first performed correlation analysis between 22 immune cell infiltration fraction and our risk score. Results revealed that CD4 memory resting T cells (*P* = 0.007) and CD4 memory activated T cells (*P* = 0.0059) were significantly higher in low-risk group (Fig. 10A–B). But the macrophages M0 cells (*P* < 0.001) and NK resting cells (*P* = 0.034) were significantly higher in high-risk group (Fig. 10C–D). In addition, we performed the correlation analysis between expression of six representative immune checkpoints and risk score. The expression level of CD274 (*P* = 0.032), HAVCR2 (*P* < 0.001) (Fig. 10E–F) and PDCD1LG2 (*P* = 0.22) (Fig. S4A) was higher in high-risk group than low-risk group. However, the expression of CTLA4 (*P* = 0.048), IDO1 (*P* = 0.017) (Fig. 10G–H) and PDCD1 (*P* = 0.085) (Fig. S4B) was lower in high- risk group. Different relationships among immune checkpoints may reflect the non-negligible tumor heterogeneity, and they may serve as a reference for future immune checkpoint inhibition treatment.

## Discussion

As the world's population ages, the incident rate of COAD is increasing globally. Genetic and epigenetic alteration, smoking and alcohol consumption, dietary factors, and inflammatory bowel disease are all contributory factors to the development of COAD. Current COAD treatment consists primarily of surgical resection and chemotherapy, but their ineffectiveness exemplifies the need for novel approaches [27]. Due to a lack of early diagnostic tools, the majority of patients are diagnosed at advanced stages of disease. As a result, many patients miss the optimal window for curative surgical treatment [28, 29]. Although numerous studies have addressed the diagnosis and treatment of COAD in the past, no meaningful breakthroughs have been made. Resultantly, establishing a reliable model for early diagnosis and prognosis prediction in COAD is paramount. The use of such a model could accurately and promptly assess the outcomes of treatment and offer recommendations for additional treatment [30]. Chen et al. and Zuo et al. [31, 32] published the COAD prognostic model of transcriptome characteristic genes, which describes the construction of prognostic models for patients. Nonetheless, the role of mitochondria-related genes in COAD has yet to be explored.

As an indispensable intracellular organelle of eukaryotes, mitochondrial function plays a crucial role in many cellular processes [33]. Mitochondria serve as a metabolic hub to regulate the metabolic process and provide energy for cell growth, differentiation, and apoptosis. It has been proven that mitochondrial dysfunction affects the occurrence and development of cancer [34]. Some biological processes related to cancer, including tumor formation, development, invasion, metastasis, and drug resistance, are dependent on mitochondria [35, 36]. Since the metabolic process in tumors is frequently changed, mitochondrial-related genes have been investigated as a potential cancer therapy target in a number of recent studies [37, 38]. Differential expression of mitochondria-related genes is associated with occurrence and metastasis of breast cancer [39], as well as the invasive phenotype of osteosarcoma [40], according to studies. During tumor initiation and metastasis, the metabolism is reprogrammed, and this reprogramming is largely dependent on mitochondria [41].

Furthermore, there are a number of studies focus on mechanisms of mitochondrial-related genes and designing corresponding drugs and inhibitors for COAD treatments. Growing tumors will quickly exceed the size that diffusion provides an adequate supply of oxygen, leading to tumor hypoxia and transition to glycolysis. This switch is caused by an important factor, hypoxia-inducible transcription factor 1α (HIF-1α), which determines metabolic fate of COAD. Downregulation of MPC1 and MPC2 has been reported in COAD, which is associated with poor prognosis [42]. Moreover, a series of researches found the role of mitochondrial oxidative phosphorylation (OXPHOS) in COAD. Increased mitochondrial DNA copy number in COAD is connected to higher proliferation and lower apoptosis by mitochondrial OXPHOS [43]. With the in-depth understanding of these metabolic processes and mitochondrial genes, recent studies to target cancer metabolism focus on the mitochondrial TCA and OXPHOS to block the aerobic glycolysis in tumor cells. A large number of drugs are under study to target mitochondria and mitochondrial function, such as metformin, 3-bromopyruvate or 2-deoxyglucose [44, 45]. Notably, most studies have explored a single mitochondrial-related gene or an associated signaling pathway in tumor formation, invasion, metastasis, and its relationship with the prognosis of cancer. In present study, the complicated biological process of mitochondria has been paid attention, and the utilization of mitochondria-related gene sets will be more reliable and can effectively judge the survival and prognosis of COAD.

Furthermore, a number of studies concentrate on the mechanisms of mitochondrial-related genes and the development of drugs and inhibitors for COAD treatments. Tumors will quickly outgrow the size at which diffusion can provide an adequate supply of oxygen, leading to tumor hypoxia and the transition to glycolysis. Hypoxia-inducible transcription factor 1α (HIF-1α) is responsible for this switch, resulting in up-regulation of several genes to avoid hypoxic stress and activate pyruvate dehydrogenase kinase (PDK) to inhibit mitochondrial metabolism [46, 47]. In COAD, HIF1α expression is associated with cancer-specific death, recurrence, vascular invasion and chemoresistance [48]. In addition, the mitochondrial pyruvate carrier (MPC), consisting of MPC1 and MPC2 subunits, becomes another pivotal factor in determining the metabolic fate of COAD [49]. Since MPC is responsible for mitochondrial pyruvate uptake, it causes oxidation in the tricarboxylic acid (TCA) cycle subsequently. MPC1 and MPC2 deletion or downregulation has been reported in COAD, which is associated with poor prognosis [50]. In addition, variety of studies have identified the effect of mitochondrial oxidative phosphorylation (OXPHOS) in COAD. Increased mitochondrial DNA copy number in COAD correlates with increased proliferation and apoptosis inhibition by mitochondrial OXPHOS [51]. With the in-depth understanding of these metabolic processes and mitochondrial genes, recent studies targeting cancer metabolism have been focusing on the mitochondrial TCA and OXPHOS to block the aerobic glycolysis in tumor cells. Numerous drugs targeting mitochondria and mitochondrial function are being investigated, such as 3-bromopyruvate, metformin or 2-deoxyglucose [44, 45]. Notably, the majority of studies have centered on a single mitochondrial-related gene or signaling pathway in tumor formation, progression and its association with cancer survival. The complex biological process of mitochondria has been considered in our work, and the utilization of nuclear mitochondria-related gene sets will be more reliable and can effectively estimate the survival status of COAD.

Many prognostic patterns according to mitochondrial-related genes have been clarified for certain cancers, including bladder, prostate, liver, and lung cancer [52–55]. However, no research has been reported in COAD. At the beginning of our research, we conducted a gene signature prediction model according to mitochondrial-related genes in COAD. Several bioinformatics instruments were used to analyze COAD sample transcriptome sequencing data. We discovered that 88 genes were up-regulated and 99 were down-regulated in COAD tissue samples compared to normal tissue by leveraging the human mitochondria-related gene library MitoMiner V4.0 [33]. The identified DEGs are closely correlated with mitochondrial dysfunction and metabolic processes during the development of COAD. GO enrichment analysis revealed genetic variations in nine biological pathways related to cancer, including ROS generation, nucleic acids, amino acid metabolism, and dicarboxylic acid metabolism [56]. These biological processes are consistent with the characteristics of tumor cells and are primarily associated with unrestricted cell proliferation [57], indicating that mitochondria-related genes is closely connected to the carcinogenesis of COAD. Differential expression of mitochondria-related genes primarily affects fatty acid metabolism and amino acid metabolism pathways, which are closely related to the metabolic adaptation of tumors and the metastasis of COAD [58], as indicated by the KEGG pathway maps. These metabolic changes are essential for tumor growth in an unfavorable tumor microenvironment and for the development and maintenance of cancer cell metastasis [59]. Numerous studies have recently proposed the "lipolytic phenotype" of cancer; fatty acid metabolism is also reprogrammed in cancer-related immune cells [60], contributing to immune suppression and promoting the tumor microenvironment, making it a potential target of immunotherapy [61].

In our present study, differentially expressed mitochondria-related genes and prognostic correlation analysis were utilized to develop a prediction model for eight key genes. We discovered that the prediction model was able to effectively stratify patients based on survival, with high-risk group exhibiting worse OS than low-risk group. ROC and independent prognosis analysis suggested that the predictive pattern could be applied as an independent risk factor for patient prognosis and had a high predictive value for patient prognosis. Clinical staging, TNM staging, and histological grading continue to be the most frequently used tools for prognostic prediction and treatment strategies in COAD patients [62] at present. However, the heterogeneity of COAD makes it challenging to improve the treatment efficacy of COAD and make decisions for doctors regarding the therapy of COAD patients [29]. A prognostic nomogram was developed in the present study, which had the advantage of overcoming COAD heterogeneity, and may lead to inaccurate prognosis prediction in COAD patients. In contrast, OS had greater AUC values at 1, 3, and 5 years, indicating that the newly constructed nomogram was credible. Through gene correlation analysis, it was discovered that CDC25C and P4HA1 may be key genes to target in COAD patients [63–66], as they are associated with the metabolism, cell cycle, and progression of tumors. Therefore, CDC25C and P4HA1 have the potential to serve as biomarkers for COAD patients and contribute to the decision-making process regarding colon cancer treatment. The novel genes including KCNJ11 NOL3, P4HA1, and QSOX2 were overlooked in COAD in the past; the correlation among these genes and COAD prognosis has been inadequately defined and requires further investigation. Our findings demonstrate the pioneering prognostic value of our model and offer a novel pathogenesis and prognostic mechanism for COAD.

Our study still has certain limitations. Even though we validated our signature model based on public GEO datasets, it is worth further validating with prospective clinical samples and local cohort data in the future. Additionally, although our study demonstrated a potential association between risk scores and tumor microenvironment or clinical characteristics that may influence clinical management decisions in patients with COAD, the validation of immune checkpoint inhibitors and patient-targeted therapies requires further research. The potential regulatory mechanisms in vivo or in vitro also need further research to explore in depth.

## Conclusion

This study represents the first effort to discover polygenic markers of mitochondrial-related genes assess potential function of these genes during the carcinogenesis of COAD patients. In addition, a robust risk score tool based on the expression profile of mitochondrial-related genes was developed to prompt COAD patients’ prognosis. Furthermore, the prognostic nomogram and mitochondrial-related gene signature were shown to have clinical applicability. In addition, the analysis of clinical and histopathological features, which bodes well for patient-specific treatment and medical decision-making in the future.

## Supplementary Information

**Additional file 1. Figure S1:** Kaplan-Meier plots of another two prognostic mitochondrion-related genes signature (P > 0.05). **Figure S2:** Decision Curve Analysis for the risk score model. **Figure S3:** Correlation analysis Association between risk score and clinicopathological characteristics (P > 0.05). **Figure S4:** Correlation analysis between risk score and immune checkpoint expression of PDCD1 and PDCD1LG2. (PDF 2274 kb)

**Additional file 2.** Raw data. (ZIP 320499 kb)

**Additional file 3. Table S1:** 249 mitochondrion-related DEGs in TCGA-COAD. (XLSX 30 kb)

**Additional file 4. Table S2:** The coefficient, 95% CI and P value of each candidate gene in mitochondrial-related gene signature model by univariate and multivariate Cox regression. (XLSX 11 kb)

## Data Availability

Publicly available datasets were analyzed in this study. The datasets TCGA-COAD and corresponding clinical patient information analyzed for this study can be found in the TCGA Knowledge Base (https://portal.gdc.cancer.gov/repository, accessed on 23 September 2022). Expression profile of GSE39582 in the manuscript was downloaded from the Gene Expression Omnibus (GEO) database (https://www.ncbi.nlm.nih.gov/geo/, accessed on 23 September 2022).

## Abbreviations

COAD: Colon adenocarcinoma
TCGA: The cancer genome atlas
DEGs: Differentially expressed genes
OS: Overall survival
ROC: Receiver operating characteristic
FDR: False discovery rate
GO: Gene ontology
KEGG: Kyoto encyclopedia of genes and genomes
GSEA: Gene set enrichment analysis
AUC: Area under the curve
HR: Hazard ratio
CI: Confidence interval
