# Supplementary material for: A novel signature model based on mitochondrial-related genes for predicting survival of colon adenocarcinoma
Source: BMC Med Inform Decis Mak. 2022 Oct 22;22:277. doi: 10.1186/s12911-022-02020-3 (PMC9587559; doi:10.1186/s12911-022-02020-3)
Supplement: Supplementary file 1 — Additional file 1. Figure S1: Kaplan-Meier plots of another two prognostic mitochondrion-related genes signature (P > 0.05). Figure S2: Decision Curve Analysis for the risk score model. Figure S3: Correlation analysis Association between risk score and clinicopathological characteristics (P > 0.05). Figure S4: Correlation analysis between risk score and immune checkpoint expression of PDCD1 and PDCD1LG2. (PDF 2274 kb) [file 12911_2022_2020_MOESM1_ESM.pdf]

Figure S1

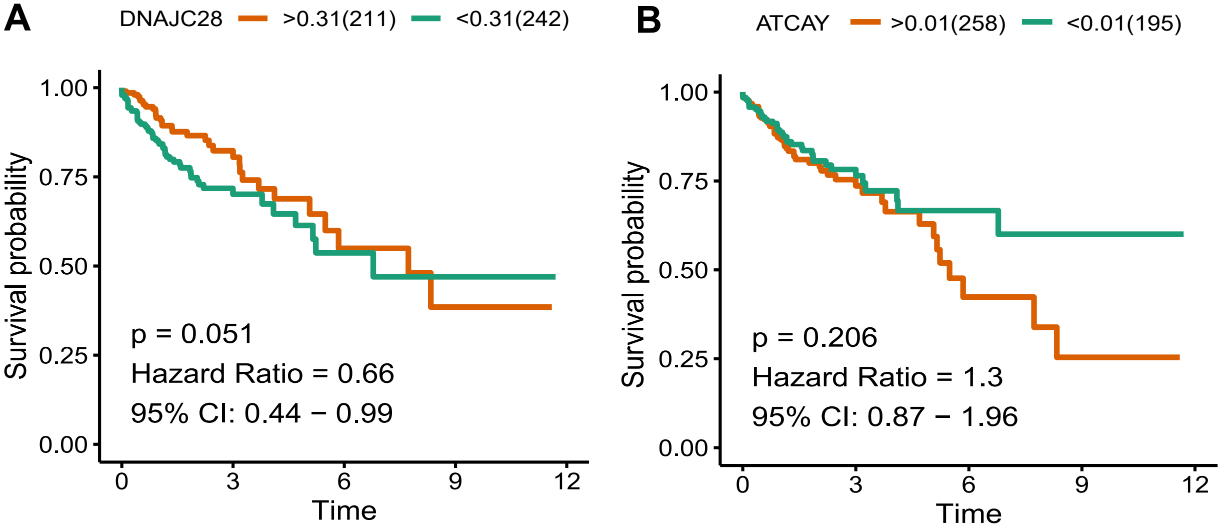

Fig S1:Kaplan-Meier plots of another two prognostic mitochondrion-related genessignature (P>0.05).

Figure S2

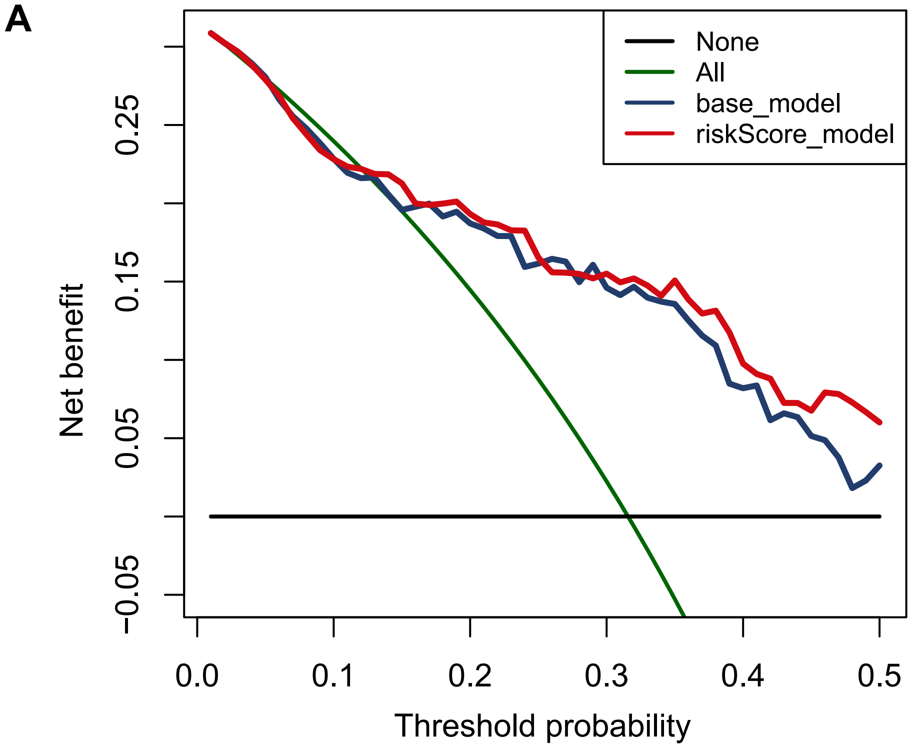

Fig S2: Decision Curve Analysis for the risk score model.

Figure S3

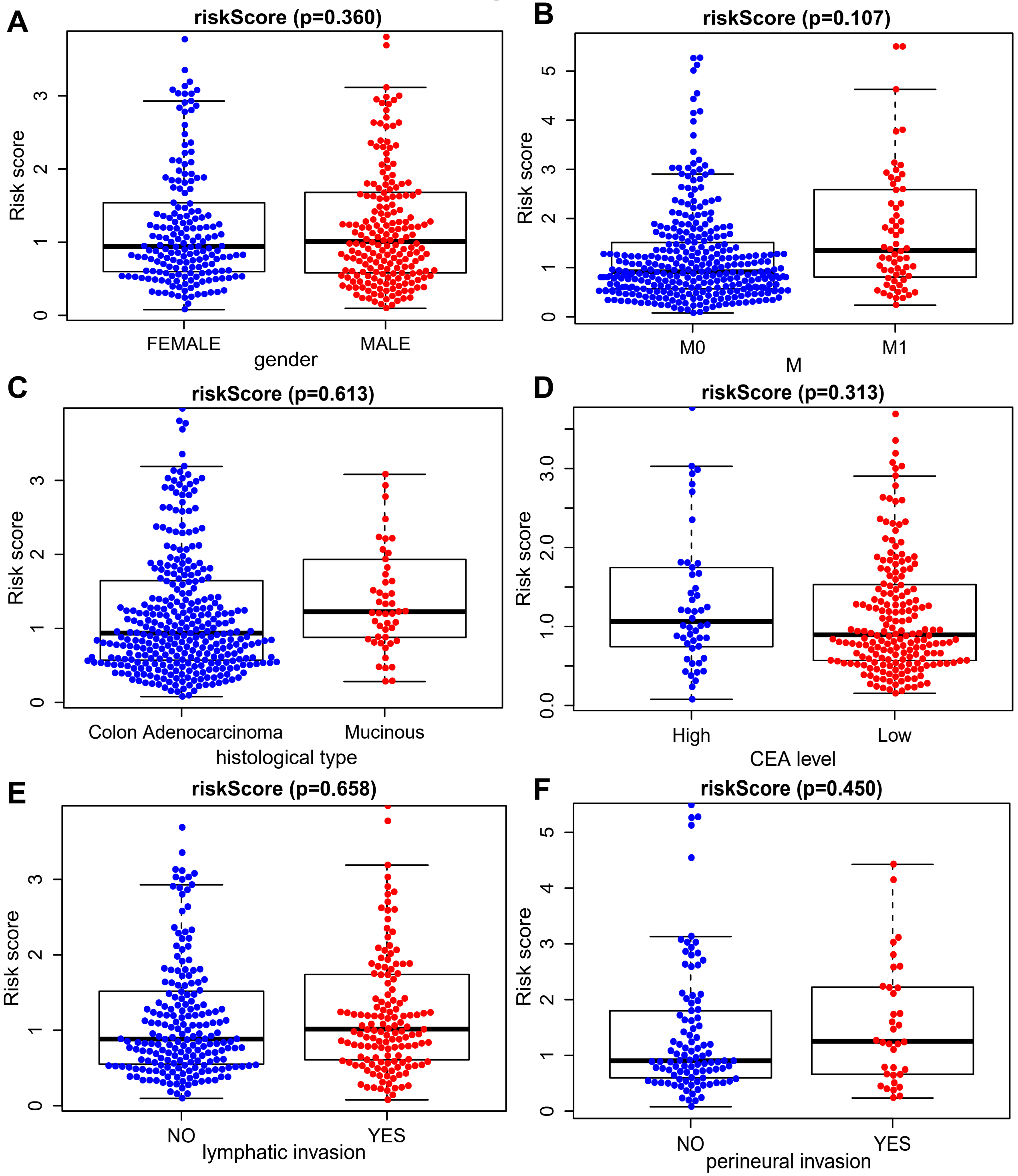

Fig S3: Correlation analysisAssociation between risk score and clinicopathological characteristics (P>0.05). (A-F): Gender, M, histological type, CEA level, lymphatic invasion and perineural invasion.

Figure S4

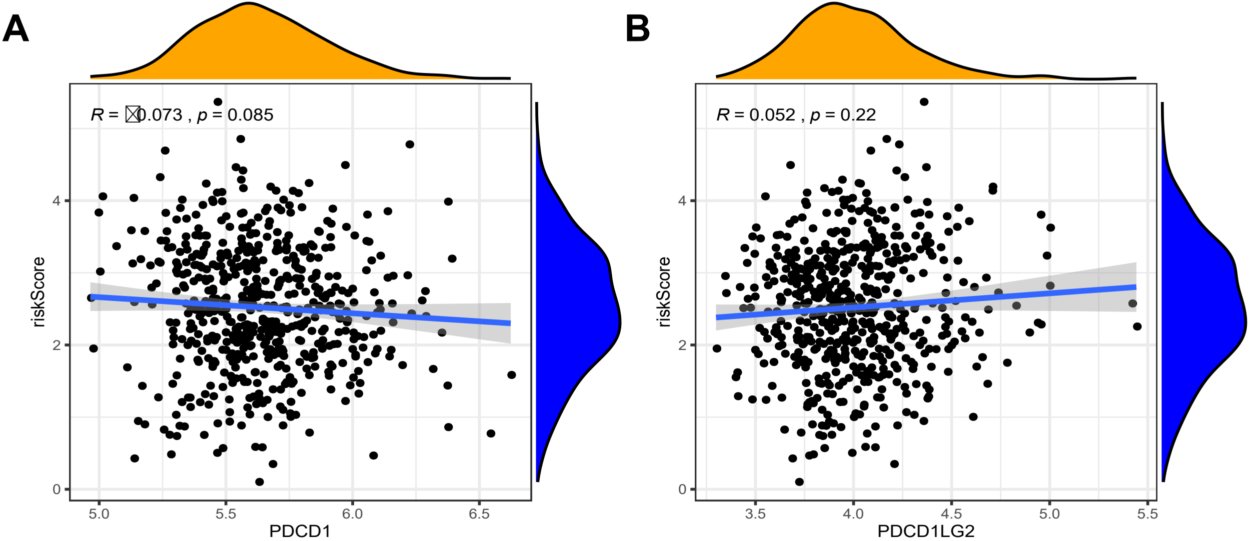

Fig S4: Correlation analysis between risk score and immune checkpoint expression of PDCD1 and PDCD1LG2.
