# Supplementary material for: A novel signature model based on mitochondrial-related genes for predicting survival of colon adenocarcinoma
Source: BMC Med Inform Decis Mak. 2022 Oct 22;22:277. doi: 10.1186/s12911-022-02020-3 (PMC9587559; doi:10.1186/s12911-022-02020-3)
Supplement: Supplementary file 2 — Additional file 2. Raw data. (ZIP 320499 kb) [file 12911_2022_2020_MOESM2_ESM.zip › Raw data/5. GSEA Result/GSEA_RESULT/GOBP_MITOCHONDRIAL_GENE_EXPRESSION.html]

Details for gene set GOBP\_MITOCHONDRIAL\_GENE\_EXPRESSION[GSEA]

|  || Dataset | input.input.cls#T\_versus\_N.input.cls#T\_versus\_N\_repos |
| Phenotype | input.cls#T\_versus\_N\_repos |
| Upregulated in class | T |
| GeneSet | GOBP\_MITOCHONDRIAL\_GENE\_EXPRESSION |
| Enrichment Score (ES) | 0.7078916 |
| Normalized Enrichment Score (NES) | 1.7720762 |
| Nominal p-value | 0.03 |
| FDR q-value | 0.017947288 |
| FWER p-Value | 0.035 |
Table: GSEA Results Summary

  

Fig 1: Enrichment plot: GOBP\_MITOCHONDRIAL\_GENE\_EXPRESSION      
 Profile of the Running ES Score & Positions of GeneSet Members on the Rank Ordered List

  

| SYMBOL | TITLE | RANK IN GENE LIST | RANK METRIC SCORE | RUNNING ES | CORE ENRICHMENT || 1 | TWNK | na | 11 | 1.518 | 0.0187 | Yes |
| 2 | PUS1 | na | 107 | 1.255 | 0.0326 | Yes |
| 3 | MRPL17 | na | 109 | 1.254 | 0.0482 | Yes |
| 4 | RPUSD4 | na | 124 | 1.239 | 0.0633 | Yes |
| 5 | CDK5RAP1 | na | 234 | 1.157 | 0.0758 | Yes |
| 6 | MALSU1 | na | 397 | 1.076 | 0.0862 | Yes |
| 7 | SHMT2 | na | 434 | 1.061 | 0.0988 | Yes |
| 8 | MRPL3 | na | 451 | 1.054 | 0.1116 | Yes |
| 9 | PNPT1 | na | 460 | 1.052 | 0.1246 | Yes |
| 10 | TBRG4 | na | 590 | 0.999 | 0.1347 | Yes |
| 11 | DAP3 | na | 626 | 0.986 | 0.1463 | Yes |
| 12 | PTCD3 | na | 633 | 0.985 | 0.1585 | Yes |
| 13 | MTG2 | na | 709 | 0.963 | 0.1691 | Yes |
| 14 | TRIT1 | na | 717 | 0.960 | 0.1809 | Yes |
| 15 | MRPS23 | na | 827 | 0.932 | 0.1905 | Yes |
| 16 | EARS2 | na | 832 | 0.931 | 0.2021 | Yes |
| 17 | MRPL36 | na | 889 | 0.917 | 0.2125 | Yes |
| 18 | YARS2 | na | 894 | 0.917 | 0.2238 | Yes |
| 19 | MRPL30 | na | 1013 | 0.890 | 0.2327 | Yes |
| 20 | MRPL37 | na | 1053 | 0.881 | 0.2430 | Yes |
| 21 | RPUSD3 | na | 1215 | 0.848 | 0.2506 | Yes |
| 22 | MRPL9 | na | 1260 | 0.839 | 0.2603 | Yes |
| 23 | MRPL58 | na | 1277 | 0.835 | 0.2704 | Yes |
| 24 | MRPL57 | na | 1292 | 0.833 | 0.2805 | Yes |
| 25 | TARS2 | na | 1364 | 0.822 | 0.2894 | Yes |
| 26 | TFB1M | na | 1391 | 0.816 | 0.2991 | Yes |
| 27 | MRPL4 | na | 1428 | 0.810 | 0.3086 | Yes |
| 28 | GADD45GIP1 | na | 1488 | 0.799 | 0.3174 | Yes |
| 29 | MRPL50 | na | 1510 | 0.794 | 0.3270 | Yes |
| 30 | HSD17B10 | na | 1546 | 0.789 | 0.3361 | Yes |
| 31 | GATC | na | 1560 | 0.785 | 0.3457 | Yes |
| 32 | FASTKD2 | na | 1713 | 0.764 | 0.3524 | Yes |
| 33 | MRPS26 | na | 1872 | 0.742 | 0.3588 | Yes |
| 34 | UQCC1 | na | 1940 | 0.732 | 0.3667 | Yes |
| 35 | MRPS2 | na | 2123 | 0.708 | 0.3722 | Yes |
| 36 | MRPS12 | na | 2201 | 0.700 | 0.3795 | Yes |
| 37 | SARS2 | na | 2316 | 0.686 | 0.3860 | Yes |
| 38 | TRMT5 | na | 2393 | 0.677 | 0.3931 | Yes |
| 39 | MRPS34 | na | 2419 | 0.675 | 0.4010 | Yes |
| 40 | MRPS27 | na | 2449 | 0.671 | 0.4088 | Yes |
| 41 | MTRF1 | na | 2611 | 0.651 | 0.4140 | Yes |
| 42 | MRPS30 | na | 2727 | 0.637 | 0.4199 | Yes |
| 43 | TRMT10C | na | 2763 | 0.633 | 0.4271 | Yes |
| 44 | UQCC2 | na | 2802 | 0.630 | 0.4343 | Yes |
| 45 | TFB2M | na | 2858 | 0.623 | 0.4410 | Yes |
| 46 | ERAL1 | na | 3003 | 0.610 | 0.4460 | Yes |
| 47 | MTG1 | na | 3077 | 0.604 | 0.4522 | Yes |
| 48 | MRPS17 | na | 3086 | 0.603 | 0.4596 | Yes |
| 49 | TRNT1 | na | 3091 | 0.603 | 0.4670 | Yes |
| 50 | RCC1L | na | 3242 | 0.590 | 0.4716 | Yes |
| 51 | TRUB2 | na | 3257 | 0.589 | 0.4787 | Yes |
| 52 | RMND1 | na | 3283 | 0.586 | 0.4855 | Yes |
| 53 | MRPL52 | na | 3321 | 0.584 | 0.4921 | Yes |
| 54 | TSFM | na | 3381 | 0.579 | 0.4983 | Yes |
| 55 | LRPPRC | na | 3389 | 0.579 | 0.5054 | Yes |
| 56 | MRPL53 | na | 3474 | 0.572 | 0.5110 | Yes |
| 57 | MRPL55 | na | 3478 | 0.572 | 0.5180 | Yes |
| 58 | MRPS31 | na | 3553 | 0.565 | 0.5237 | Yes |
| 59 | MPV17L2 | na | 3579 | 0.563 | 0.5303 | Yes |
| 60 | C1QBP | na | 3584 | 0.562 | 0.5372 | Yes |
| 61 | MRRF | na | 3714 | 0.553 | 0.5417 | Yes |
| 62 | MRPL48 | na | 3905 | 0.538 | 0.5450 | Yes |
| 63 | POLRMT | na | 3924 | 0.536 | 0.5513 | Yes |
| 64 | MRPL45 | na | 3948 | 0.535 | 0.5576 | Yes |
| 65 | TRMT61B | na | 3977 | 0.532 | 0.5637 | Yes |
| 66 | MRPL19 | na | 4052 | 0.527 | 0.5689 | Yes |
| 67 | MRPL42 | na | 4106 | 0.524 | 0.5745 | Yes |
| 68 | SUPV3L1 | na | 4126 | 0.523 | 0.5806 | Yes |
| 69 | MRPL47 | na | 4380 | 0.506 | 0.5824 | Yes |
| 70 | MRPS5 | na | 4391 | 0.505 | 0.5885 | Yes |
| 71 | MRPS15 | na | 4514 | 0.497 | 0.5924 | Yes |
| 72 | MRPL51 | na | 4662 | 0.490 | 0.5959 | Yes |
| 73 | MRPL15 | na | 4705 | 0.488 | 0.6012 | Yes |
| 74 | MRPS10 | na | 4860 | 0.478 | 0.6043 | Yes |
| 75 | MRPL24 | na | 4882 | 0.477 | 0.6099 | Yes |
| 76 | MRPL22 | na | 5025 | 0.470 | 0.6132 | Yes |
| 77 | ELAC2 | na | 5098 | 0.466 | 0.6177 | Yes |
| 78 | LARS2 | na | 5170 | 0.462 | 0.6221 | Yes |
| 79 | MRPL23 | na | 5251 | 0.457 | 0.6264 | Yes |
| 80 | MRPS35 | na | 5271 | 0.456 | 0.6317 | Yes |
| 81 | TFAM | na | 5413 | 0.449 | 0.6347 | Yes |
| 82 | TRMT10B | na | 5467 | 0.446 | 0.6393 | Yes |
| 83 | MRPS18B | na | 5557 | 0.441 | 0.6432 | Yes |
| 84 | NOA1 | na | 5579 | 0.440 | 0.6483 | Yes |
| 85 | QRSL1 | na | 5617 | 0.438 | 0.6531 | Yes |
| 86 | MRPL16 | na | 5669 | 0.436 | 0.6575 | Yes |
| 87 | MRPL18 | na | 5760 | 0.432 | 0.6613 | Yes |
| 88 | WARS2 | na | 5970 | 0.423 | 0.6628 | Yes |
| 89 | MRPL11 | na | 6123 | 0.416 | 0.6652 | Yes |
| 90 | MRPL32 | na | 6283 | 0.409 | 0.6674 | Yes |
| 91 | MTIF3 | na | 6376 | 0.405 | 0.6708 | Yes |
| 92 | DARS2 | na | 6406 | 0.403 | 0.6753 | Yes |
| 93 | MRPL13 | na | 6432 | 0.402 | 0.6798 | Yes |
| 94 | MRPL38 | na | 6761 | 0.389 | 0.6787 | Yes |
| 95 | MRPL40 | na | 6986 | 0.381 | 0.6794 | Yes |
| 96 | MTIF2 | na | 7029 | 0.379 | 0.6833 | Yes |
| 97 | MRPL21 | na | 7032 | 0.379 | 0.6880 | Yes |
| 98 | MRPL28 | na | 7051 | 0.378 | 0.6924 | Yes |
| 99 | MRPL14 | na | 7171 | 0.374 | 0.6949 | Yes |
| 100 | TUFM | na | 7237 | 0.372 | 0.6983 | Yes |
| 101 | MRPL20 | na | 7344 | 0.367 | 0.7010 | Yes |
| 102 | AARS2 | na | 7678 | 0.355 | 0.6994 | Yes |
| 103 | MRPS25 | na | 7809 | 0.350 | 0.7014 | Yes |
| 104 | MRPS21 | na | 7837 | 0.349 | 0.7052 | Yes |
| 105 | MRPL10 | na | 7928 | 0.346 | 0.7079 | Yes |
| 106 | MRPS16 | na | 8559 | 0.325 | 0.7005 | No |
| 107 | MTERF2 | na | 8615 | 0.323 | 0.7035 | No |
| 108 | NDUFA7 | na | 8824 | 0.317 | 0.7037 | No |
| 109 | MRPS14 | na | 9236 | 0.304 | 0.7000 | No |
| 110 | MRPL12 | na | 9338 | 0.301 | 0.7019 | No |
| 111 | MTO1 | na | 9576 | 0.294 | 0.7013 | No |
| 112 | MRPL2 | na | 9586 | 0.294 | 0.7048 | No |
| 113 | GFM1 | na | 9888 | 0.286 | 0.7029 | No |
| 114 | IARS2 | na | 9940 | 0.285 | 0.7055 | No |
| 115 | TEFM | na | 10154 | 0.279 | 0.7051 | No |
| 116 | RARS2 | na | 10708 | 0.265 | 0.6984 | No |
| 117 | MRPS33 | na | 11282 | 0.251 | 0.6911 | No |
| 118 | CHCHD1 | na | 11466 | 0.247 | 0.6909 | No |
| 119 | TRMT10A | na | 11792 | 0.240 | 0.6880 | No |
| 120 | FASTKD5 | na | 12157 | 0.233 | 0.6843 | No |
| 121 | ALKBH1 | na | 12618 | 0.224 | 0.6787 | No |
| 122 | MRPL1 | na | 12678 | 0.223 | 0.6804 | No |
| 123 | MRPL43 | na | 13207 | 0.213 | 0.6735 | No |
| 124 | FASTKD3 | na | 13305 | 0.211 | 0.6743 | No |
| 125 | MRPS24 | na | 13348 | 0.210 | 0.6762 | No |
| 126 | MRPS28 | na | 13365 | 0.210 | 0.6785 | No |
| 127 | MRPL49 | na | 14301 | 0.196 | 0.6640 | No |
| 128 | MRPS22 | na | 14467 | 0.193 | 0.6634 | No |
| 129 | GFM2 | na | 14599 | 0.191 | 0.6634 | No |
| 130 | NGRN | na | 14792 | 0.189 | 0.6623 | No |
| 131 | MTERF4 | na | 16097 | 0.174 | 0.6408 | No |
| 132 | MRPS9 | na | 16951 | 0.166 | 0.6273 | No |
| 133 | MRPL33 | na | 21517 | 0.131 | 0.5461 | No |
| 134 | SLC25A33 | na | 22895 | 0.119 | 0.5226 | No |
| 135 | MRPL41 | na | 23157 | 0.117 | 0.5194 | No |
| 136 | MRPL39 | na | 23652 | 0.112 | 0.5118 | No |
| 137 | MRPS7 | na | 25988 | 0.093 | 0.4706 | No |
| 138 | MRPL27 | na | 26088 | 0.093 | 0.4699 | No |
| 139 | MRPL44 | na | 26207 | 0.092 | 0.4689 | No |
| 140 | AURKAIP1 | na | 26406 | 0.090 | 0.4665 | No |
| 141 | MRPL54 | na | 31502 | 0.052 | 0.3746 | No |
| 142 | MRPS18A | na | 32074 | 0.048 | 0.3649 | No |
| 143 | MRPS6 | na | 35910 | 0.022 | 0.2956 | No |
| 144 | METTL4 | na | 36539 | 0.019 | 0.2844 | No |
| 145 | FOXO3 | na | 37498 | 0.015 | 0.2672 | No |
| 146 | GATB | na | 38666 | 0.011 | 0.2462 | No |
| 147 | MRPS18C | na | 44000 | -0.016 | 0.1496 | No |
| 148 | OXA1L | na | 45395 | -0.053 | 0.1250 | No |
| 149 | MRPL46 | na | 45726 | -0.064 | 0.1198 | No |
| 150 | PRKAA1 | na | 45733 | -0.064 | 0.1204 | No |
| 151 | COA3 | na | 45972 | -0.072 | 0.1170 | No |
| 152 | MTERF1 | na | 48001 | -0.151 | 0.0821 | No |
| 153 | NSUN3 | na | 48104 | -0.156 | 0.0822 | No |
| 154 | MTRF1L | na | 48612 | -0.181 | 0.0752 | No |
| 155 | MRPL34 | na | 49149 | -0.211 | 0.0681 | No |
| 156 | TACO1 | na | 50275 | -0.283 | 0.0512 | No |
| 157 | MRPS11 | na | 51927 | -0.428 | 0.0266 | No |
| 158 | MRPS36 | na | 52542 | -0.489 | 0.0216 | No |
| 159 | CHCHD10 | na | 52698 | -0.505 | 0.0250 | No |
| 160 | MRPL35 | na | 54294 | -0.809 | 0.0061 | No |
| 161 | PPARGC1B | na | 54552 | -0.923 | 0.0130 | No |
Table: GSEA details [plain text format]

  

Fig 2: GOBP\_MITOCHONDRIAL\_GENE\_EXPRESSION      
 Blue-Pink O' Gram in the Space of the Analyzed GeneSet

  

Fig 3: GOBP\_MITOCHONDRIAL\_GENE\_EXPRESSION: Random ES distribution      
 Gene set null distribution of ES for **GOBP\_MITOCHONDRIAL\_GENE\_EXPRESSION**

  
