# Supplementary material for: A novel signature model based on mitochondrial-related genes for predicting survival of colon adenocarcinoma
Source: BMC Med Inform Decis Mak. 2022 Oct 22;22:277. doi: 10.1186/s12911-022-02020-3 (PMC9587559; doi:10.1186/s12911-022-02020-3)
Supplement: Supplementary file 2 — Additional file 2. Raw data. (ZIP 320499 kb) [file 12911_2022_2020_MOESM2_ESM.zip › Raw data/5. GSEA Result/GSEA_RESULT/GOBP_MITOCHONDRIAL_GENOME_MAINTENANCE.html]

Details for gene set GOBP\_MITOCHONDRIAL\_GENOME\_MAINTENANCE[GSEA]

|  || Dataset | input.input.cls#T\_versus\_N.input.cls#T\_versus\_N\_repos |
| Phenotype | input.cls#T\_versus\_N\_repos |
| Upregulated in class | T |
| GeneSet | GOBP\_MITOCHONDRIAL\_GENOME\_MAINTENANCE |
| Enrichment Score (ES) | 0.58461004 |
| Normalized Enrichment Score (NES) | 1.6818372 |
| Nominal p-value | 0.015267176 |
| FDR q-value | 0.024674293 |
| FWER p-Value | 0.064 |
Table: GSEA Results Summary

  

Fig 1: Enrichment plot: GOBP\_MITOCHONDRIAL\_GENOME\_MAINTENANCE      
 Profile of the Running ES Score & Positions of GeneSet Members on the Rank Ordered List

  

| SYMBOL | TITLE | RANK IN GENE LIST | RANK METRIC SCORE | RUNNING ES | CORE ENRICHMENT || 1 | PIF1 | na | 666 | 0.977 | 0.0681 | Yes |
| 2 | MPV17 | na | 808 | 0.936 | 0.1425 | Yes |
| 3 | DNA2 | na | 892 | 0.917 | 0.2163 | Yes |
| 4 | POLG2 | na | 1170 | 0.855 | 0.2815 | Yes |
| 5 | LIG3 | na | 1171 | 0.855 | 0.3517 | Yes |
| 6 | LONP1 | na | 1345 | 0.824 | 0.4163 | Yes |
| 7 | MGME1 | na | 1451 | 0.806 | 0.4806 | Yes |
| 8 | TP53 | na | 2831 | 0.626 | 0.5070 | Yes |
| 9 | STOX1 | na | 3611 | 0.560 | 0.5389 | Yes |
| 10 | PARP1 | na | 3623 | 0.559 | 0.5846 | Yes |
| 11 | SLC25A36 | na | 7998 | 0.343 | 0.5336 | No |
| 12 | TOP3A | na | 11929 | 0.237 | 0.4820 | No |
| 13 | TYMP | na | 16691 | 0.168 | 0.4096 | No |
| 14 | SLC25A33 | na | 22895 | 0.119 | 0.3071 | No |
| 15 | OPA1 | na | 22907 | 0.119 | 0.3167 | No |
| 16 | PRIMPOL | na | 46934 | -0.107 | -0.1094 | No |
| 17 | AKT3 | na | 49566 | -0.236 | -0.1377 | No |
| 18 | MEF2A | na | 51767 | -0.412 | -0.1436 | No |
| 19 | SLC25A4 | na | 52264 | -0.462 | -0.1146 | No |
| 20 | PPARGC1A | na | 54725 | -1.004 | -0.0767 | No |
| 21 | SESN2 | na | 54802 | -1.053 | 0.0084 | No |
Table: GSEA details [plain text format]

  

Fig 2: GOBP\_MITOCHONDRIAL\_GENOME\_MAINTENANCE      
 Blue-Pink O' Gram in the Space of the Analyzed GeneSet

  

Fig 3: GOBP\_MITOCHONDRIAL\_GENOME\_MAINTENANCE: Random ES distribution      
 Gene set null distribution of ES for **GOBP\_MITOCHONDRIAL\_GENOME\_MAINTENANCE**

  
