# Supplementary material for: A novel signature model based on mitochondrial-related genes for predicting survival of colon adenocarcinoma
Source: BMC Med Inform Decis Mak. 2022 Oct 22;22:277. doi: 10.1186/s12911-022-02020-3 (PMC9587559; doi:10.1186/s12911-022-02020-3)
Supplement: Supplementary file 2 — Additional file 2. Raw data. (ZIP 320499 kb) [file 12911_2022_2020_MOESM2_ESM.zip › Raw data/5. GSEA Result/GSEA_RESULT/GOBP_MITOCHONDRIAL_RNA_METABOLIC_PROCESS.html]

Details for gene set GOBP\_MITOCHONDRIAL\_RNA\_METABOLIC\_PROCESS[GSEA]

|  || Dataset | input.input.cls#T\_versus\_N.input.cls#T\_versus\_N\_repos |
| Phenotype | input.cls#T\_versus\_N\_repos |
| Upregulated in class | T |
| GeneSet | GOBP\_MITOCHONDRIAL\_RNA\_METABOLIC\_PROCESS |
| Enrichment Score (ES) | 0.7097431 |
| Normalized Enrichment Score (NES) | 1.8745604 |
| Nominal p-value | 0.012269938 |
| FDR q-value | 0.007984204 |
| FWER p-Value | 0.01 |
Table: GSEA Results Summary

  

Fig 1: Enrichment plot: GOBP\_MITOCHONDRIAL\_RNA\_METABOLIC\_PROCESS      
 Profile of the Running ES Score & Positions of GeneSet Members on the Rank Ordered List

  

| SYMBOL | TITLE | RANK IN GENE LIST | RANK METRIC SCORE | RUNNING ES | CORE ENRICHMENT || 1 | TWNK | na | 11 | 1.518 | 0.0628 | Yes |
| 2 | PUS1 | na | 107 | 1.255 | 0.1133 | Yes |
| 3 | CDK5RAP1 | na | 234 | 1.157 | 0.1590 | Yes |
| 4 | PNPT1 | na | 460 | 1.052 | 0.1986 | Yes |
| 5 | TBRG4 | na | 590 | 0.999 | 0.2378 | Yes |
| 6 | TRIT1 | na | 717 | 0.960 | 0.2754 | Yes |
| 7 | EARS2 | na | 832 | 0.931 | 0.3120 | Yes |
| 8 | YARS2 | na | 894 | 0.917 | 0.3490 | Yes |
| 9 | TARS2 | na | 1364 | 0.822 | 0.3746 | Yes |
| 10 | TFB1M | na | 1391 | 0.816 | 0.4081 | Yes |
| 11 | HSD17B10 | na | 1546 | 0.789 | 0.4380 | Yes |
| 12 | SARS2 | na | 2316 | 0.686 | 0.4526 | Yes |
| 13 | TRMT5 | na | 2393 | 0.677 | 0.4794 | Yes |
| 14 | TRMT10C | na | 2763 | 0.633 | 0.4990 | Yes |
| 15 | TFB2M | na | 2858 | 0.623 | 0.5232 | Yes |
| 16 | TRNT1 | na | 3091 | 0.603 | 0.5441 | Yes |
| 17 | LRPPRC | na | 3389 | 0.579 | 0.5627 | Yes |
| 18 | POLRMT | na | 3924 | 0.536 | 0.5753 | Yes |
| 19 | TRMT61B | na | 3977 | 0.532 | 0.5965 | Yes |
| 20 | SUPV3L1 | na | 4126 | 0.523 | 0.6155 | Yes |
| 21 | ELAC2 | na | 5098 | 0.466 | 0.6173 | Yes |
| 22 | TFAM | na | 5413 | 0.449 | 0.6302 | Yes |
| 23 | TRMT10B | na | 5467 | 0.446 | 0.6478 | Yes |
| 24 | WARS2 | na | 5970 | 0.423 | 0.6563 | Yes |
| 25 | GRSF1 | na | 6333 | 0.407 | 0.6666 | Yes |
| 26 | DARS2 | na | 6406 | 0.403 | 0.6821 | Yes |
| 27 | SLIRP | na | 6745 | 0.389 | 0.6921 | Yes |
| 28 | AARS2 | na | 7678 | 0.355 | 0.6900 | Yes |
| 29 | PDE12 | na | 7692 | 0.354 | 0.7045 | Yes |
| 30 | MTERF2 | na | 8615 | 0.323 | 0.7012 | Yes |
| 31 | MRPL12 | na | 9338 | 0.301 | 0.7006 | Yes |
| 32 | MTO1 | na | 9576 | 0.294 | 0.7086 | Yes |
| 33 | TEFM | na | 10154 | 0.279 | 0.7097 | Yes |
| 34 | TRMT10A | na | 11792 | 0.240 | 0.6901 | No |
| 35 | FASTKD5 | na | 12157 | 0.233 | 0.6931 | No |
| 36 | MTERF4 | na | 16097 | 0.174 | 0.6290 | No |
| 37 | SLC25A33 | na | 22895 | 0.119 | 0.5109 | No |
| 38 | METTL4 | na | 36539 | 0.019 | 0.2646 | No |
| 39 | FOXO3 | na | 37498 | 0.015 | 0.2479 | No |
| 40 | PRKAA1 | na | 45733 | -0.064 | 0.1015 | No |
| 41 | FASTKD1 | na | 47599 | -0.133 | 0.0732 | No |
| 42 | MTERF1 | na | 48001 | -0.151 | 0.0722 | No |
| 43 | CHCHD10 | na | 52698 | -0.505 | 0.0082 | No |
| 44 | PPARGC1B | na | 54552 | -0.923 | 0.0129 | No |
Table: GSEA details [plain text format]

  

Fig 2: GOBP\_MITOCHONDRIAL\_RNA\_METABOLIC\_PROCESS      
 Blue-Pink O' Gram in the Space of the Analyzed GeneSet

  

Fig 3: GOBP\_MITOCHONDRIAL\_RNA\_METABOLIC\_PROCESS: Random ES distribution      
 Gene set null distribution of ES for **GOBP\_MITOCHONDRIAL\_RNA\_METABOLIC\_PROCESS**

  
