# Supplementary material for: A novel signature model based on mitochondrial-related genes for predicting survival of colon adenocarcinoma
Source: BMC Med Inform Decis Mak. 2022 Oct 22;22:277. doi: 10.1186/s12911-022-02020-3 (PMC9587559; doi:10.1186/s12911-022-02020-3)
Supplement: Supplementary file 2 — Additional file 2. Raw data. (ZIP 320499 kb) [file 12911_2022_2020_MOESM2_ESM.zip › Raw data/5. GSEA Result/GSEA_RESULT/GOBP_MITOCHONDRIAL_RNA_PROCESSING.html]

Details for gene set GOBP\_MITOCHONDRIAL\_RNA\_PROCESSING[GSEA]

|  || Dataset | input.input.cls#T\_versus\_N.input.cls#T\_versus\_N\_repos |
| Phenotype | input.cls#T\_versus\_N\_repos |
| Upregulated in class | T |
| GeneSet | GOBP\_MITOCHONDRIAL\_RNA\_PROCESSING |
| Enrichment Score (ES) | 0.8306582 |
| Normalized Enrichment Score (NES) | 1.8650794 |
| Nominal p-value | 0.0 |
| FDR q-value | 0.004931105 |
| FWER p-Value | 0.011 |
Table: GSEA Results Summary

  

Fig 1: Enrichment plot: GOBP\_MITOCHONDRIAL\_RNA\_PROCESSING      
 Profile of the Running ES Score & Positions of GeneSet Members on the Rank Ordered List

  

| SYMBOL | TITLE | RANK IN GENE LIST | RANK METRIC SCORE | RUNNING ES | CORE ENRICHMENT || 1 | PUS1 | na | 107 | 1.255 | 0.1136 | Yes |
| 2 | CDK5RAP1 | na | 234 | 1.157 | 0.2179 | Yes |
| 3 | PNPT1 | na | 460 | 1.052 | 0.3107 | Yes |
| 4 | TBRG4 | na | 590 | 0.999 | 0.4004 | Yes |
| 5 | TRIT1 | na | 717 | 0.960 | 0.4865 | Yes |
| 6 | HSD17B10 | na | 1546 | 0.789 | 0.5441 | Yes |
| 7 | TRMT5 | na | 2393 | 0.677 | 0.5912 | Yes |
| 8 | TRMT10C | na | 2763 | 0.633 | 0.6429 | Yes |
| 9 | TRNT1 | na | 3091 | 0.603 | 0.6925 | Yes |
| 10 | TRMT61B | na | 3977 | 0.532 | 0.7255 | Yes |
| 11 | SUPV3L1 | na | 4126 | 0.523 | 0.7710 | Yes |
| 12 | ELAC2 | na | 5098 | 0.466 | 0.7963 | Yes |
| 13 | TRMT10B | na | 5467 | 0.446 | 0.8307 | Yes |
| 14 | MTO1 | na | 9576 | 0.294 | 0.7834 | No |
| 15 | TRMT10A | na | 11792 | 0.240 | 0.7654 | No |
| 16 | FASTKD5 | na | 12157 | 0.233 | 0.7802 | No |
Table: GSEA details [plain text format]

  

Fig 2: GOBP\_MITOCHONDRIAL\_RNA\_PROCESSING      
 Blue-Pink O' Gram in the Space of the Analyzed GeneSet

  

Fig 3: GOBP\_MITOCHONDRIAL\_RNA\_PROCESSING: Random ES distribution      
 Gene set null distribution of ES for **GOBP\_MITOCHONDRIAL\_RNA\_PROCESSING**

  
