# Supplementary material for: A novel signature model based on mitochondrial-related genes for predicting survival of colon adenocarcinoma
Source: BMC Med Inform Decis Mak. 2022 Oct 22;22:277. doi: 10.1186/s12911-022-02020-3 (PMC9587559; doi:10.1186/s12911-022-02020-3)
Supplement: Supplementary file 2 — Additional file 2. Raw data. (ZIP 320499 kb) [file 12911_2022_2020_MOESM2_ESM.zip › Raw data/5. GSEA Result/GSEA_RESULT/GOBP_POSITIVE_REGULATION_OF_MITOCHONDRIAL_OUTER_MEMBRANE_PERMEABILIZATION_INVOLVED_IN_APOPTOTIC_SIGNALING_PATHWAY.html]

Details for gene set GOBP\_POSITIVE\_REGULATION\_OF\_MITOCHONDRIAL\_OUTER\_MEMBRANE\_PERMEABILIZATION\_INVOLVED\_IN\_APOPTOTIC\_SIGNALING\_PATHWAY[GSEA]

|  || Dataset | input.input.cls#T\_versus\_N.input.cls#T\_versus\_N\_repos |
| Phenotype | input.cls#T\_versus\_N\_repos |
| Upregulated in class | T |
| GeneSet | GOBP\_POSITIVE\_REGULATION\_OF\_MITOCHONDRIAL\_OUTER\_MEMBRANE\_PERMEABILIZATION\_INVOLVED\_IN\_APOPTOTIC\_SIGNALING\_PATHWAY |
| Enrichment Score (ES) | 0.5531045 |
| Normalized Enrichment Score (NES) | 1.6052498 |
| Nominal p-value | 0.028056113 |
| FDR q-value | 0.036870886 |
| FWER p-Value | 0.092 |
Table: GSEA Results Summary

  

Fig 1: Enrichment plot: GOBP\_POSITIVE\_REGULATION\_OF\_MITOCHONDRIAL\_OUTER\_MEMBRANE\_PERMEABILIZATION\_INVOLVED\_IN\_APOPTOTIC\_SIGNALING\_PATHWAY      
 Profile of the Running ES Score & Positions of GeneSet Members on the Rank Ordered List

  

| SYMBOL | TITLE | RANK IN GENE LIST | RANK METRIC SCORE | RUNNING ES | CORE ENRICHMENT || 1 | E2F1 | na | 498 | 1.037 | 0.0507 | Yes |
| 2 | TFDP1 | na | 517 | 1.029 | 0.1097 | Yes |
| 3 | YWHAG | na | 1097 | 0.871 | 0.1495 | Yes |
| 4 | BID | na | 1101 | 0.870 | 0.1996 | Yes |
| 5 | ZNF205 | na | 1827 | 0.749 | 0.2296 | Yes |
| 6 | BBC3 | na | 1946 | 0.731 | 0.2697 | Yes |
| 7 | NMT1 | na | 2214 | 0.698 | 0.3051 | Yes |
| 8 | TP73 | na | 2249 | 0.693 | 0.3444 | Yes |
| 9 | TP53 | na | 2831 | 0.626 | 0.3700 | Yes |
| 10 | BAX | na | 4364 | 0.507 | 0.3715 | Yes |
| 11 | GSK3B | na | 4426 | 0.503 | 0.3994 | Yes |
| 12 | PMAIP1 | na | 4646 | 0.491 | 0.4237 | Yes |
| 13 | GZMB | na | 4704 | 0.488 | 0.4508 | Yes |
| 14 | YWHAB | na | 5845 | 0.427 | 0.4548 | Yes |
| 15 | PPP3R1 | na | 5905 | 0.425 | 0.4783 | Yes |
| 16 | YWHAZ | na | 6260 | 0.410 | 0.4955 | Yes |
| 17 | BOK | na | 6809 | 0.387 | 0.5079 | Yes |
| 18 | HIP1R | na | 8199 | 0.337 | 0.5022 | Yes |
| 19 | TP53BP2 | na | 8762 | 0.318 | 0.5103 | Yes |
| 20 | YWHAE | na | 8953 | 0.313 | 0.5250 | Yes |
| 21 | YWHAQ | na | 9074 | 0.309 | 0.5406 | Yes |
| 22 | CASP8 | na | 9343 | 0.301 | 0.5531 | Yes |
| 23 | TFDP2 | na | 17898 | 0.159 | 0.4074 | No |
| 24 | YWHAH | na | 19462 | 0.147 | 0.3876 | No |
| 25 | MAPK8 | na | 19716 | 0.145 | 0.3913 | No |
| 26 | GSK3A | na | 30282 | 0.061 | 0.2036 | No |
| 27 | TP63 | na | 33348 | 0.038 | 0.1503 | No |
| 28 | PPP3CC | na | 47152 | -0.116 | -0.0930 | No |
| 29 | ATP5IF1 | na | 50427 | -0.294 | -0.1353 | No |
| 30 | SFN | na | 50742 | -0.318 | -0.1227 | No |
| 31 | CHCHD10 | na | 52698 | -0.505 | -0.1290 | No |
| 32 | PPP1R13B | na | 52787 | -0.515 | -0.1009 | No |
| 33 | BAK1 | na | 53758 | -0.670 | -0.0799 | No |
| 34 | BAD | na | 54147 | -0.768 | -0.0426 | No |
| 35 | BCL2 | na | 54853 | -1.090 | 0.0075 | No |
Table: GSEA details [plain text format]

  

Fig 2: GOBP\_POSITIVE\_REGULATION\_OF\_MITOCHONDRIAL\_OUTER\_MEMBRANE\_PERMEABILIZATION\_INVOLVED\_IN\_APOPTOTIC\_SIGNALING\_PATHWAY      
 Blue-Pink O' Gram in the Space of the Analyzed GeneSet

  

Fig 3: GOBP\_POSITIVE\_REGULATION\_OF\_MITOCHONDRIAL\_OUTER\_MEMBRANE\_PERMEABILIZATION\_INVOLVED\_IN\_APOPTOTIC\_SIGNALING\_PATHWAY: Random ES distribution      
 Gene set null distribution of ES for **GOBP\_POSITIVE\_REGULATION\_OF\_MITOCHONDRIAL\_OUTER\_MEMBRANE\_PERMEABILIZATION\_INVOLVED\_IN\_APOPTOTIC\_SIGNALING\_PATHWAY**

  
