# Supplementary material for: A novel signature model based on mitochondrial-related genes for predicting survival of colon adenocarcinoma
Source: BMC Med Inform Decis Mak. 2022 Oct 22;22:277. doi: 10.1186/s12911-022-02020-3 (PMC9587559; doi:10.1186/s12911-022-02020-3)
Supplement: Supplementary file 2 — Additional file 2. Raw data. (ZIP 320499 kb) [file 12911_2022_2020_MOESM2_ESM.zip › Raw data/5. GSEA Result/GSEA_RESULT/GOBP_PROTEIN_IMPORT_INTO_MITOCHONDRIAL_MATRIX.html]

Details for gene set GOBP\_PROTEIN\_IMPORT\_INTO\_MITOCHONDRIAL\_MATRIX[GSEA]

|  || Dataset | input.input.cls#T\_versus\_N.input.cls#T\_versus\_N\_repos |
| Phenotype | input.cls#T\_versus\_N\_repos |
| Upregulated in class | T |
| GeneSet | GOBP\_PROTEIN\_IMPORT\_INTO\_MITOCHONDRIAL\_MATRIX |
| Enrichment Score (ES) | 0.72825927 |
| Normalized Enrichment Score (NES) | 1.7507724 |
| Nominal p-value | 0.01171875 |
| FDR q-value | 0.01881582 |
| FWER p-Value | 0.041 |
Table: GSEA Results Summary

  

Fig 1: Enrichment plot: GOBP\_PROTEIN\_IMPORT\_INTO\_MITOCHONDRIAL\_MATRIX      
 Profile of the Running ES Score & Positions of GeneSet Members on the Rank Ordered List

  

| SYMBOL | TITLE | RANK IN GENE LIST | RANK METRIC SCORE | RUNNING ES | CORE ENRICHMENT || 1 | TIMM50 | na | 467 | 1.048 | 0.0792 | Yes |
| 2 | GRPEL2 | na | 545 | 1.015 | 0.1628 | Yes |
| 3 | PAM16 | na | 1172 | 0.855 | 0.2229 | Yes |
| 4 | TOMM20 | na | 1185 | 0.854 | 0.2941 | Yes |
| 5 | TOMM40 | na | 1414 | 0.813 | 0.3580 | Yes |
| 6 | TIMM17B | na | 1704 | 0.765 | 0.4167 | Yes |
| 7 | GRPEL1 | na | 1806 | 0.751 | 0.4777 | Yes |
| 8 | TIMM23 | na | 2203 | 0.700 | 0.5291 | Yes |
| 9 | TIMM44 | na | 2966 | 0.613 | 0.5665 | Yes |
| 10 | TOMM70 | na | 3674 | 0.556 | 0.6002 | Yes |
| 11 | DNAJC15 | na | 3938 | 0.535 | 0.6402 | Yes |
| 12 | TIMM17A | na | 4484 | 0.499 | 0.6721 | Yes |
| 13 | TIMM23B | na | 5071 | 0.467 | 0.7006 | Yes |
| 14 | ROMO1 | na | 6368 | 0.405 | 0.7110 | Yes |
| 15 | DNLZ | na | 7427 | 0.364 | 0.7223 | Yes |
| 16 | DNAJC19 | na | 8594 | 0.324 | 0.7283 | Yes |
| 17 | TOMM7 | na | 11673 | 0.243 | 0.6928 | No |
| 18 | TOMM20L | na | 20430 | 0.139 | 0.5460 | No |
| 19 | TIMM21 | na | 49414 | -0.226 | 0.0403 | No |
| 20 | TOMM40L | na | 54205 | -0.784 | 0.0192 | No |
Table: GSEA details [plain text format]

  

Fig 2: GOBP\_PROTEIN\_IMPORT\_INTO\_MITOCHONDRIAL\_MATRIX      
 Blue-Pink O' Gram in the Space of the Analyzed GeneSet

  

Fig 3: GOBP\_PROTEIN\_IMPORT\_INTO\_MITOCHONDRIAL\_MATRIX: Random ES distribution      
 Gene set null distribution of ES for **GOBP\_PROTEIN\_IMPORT\_INTO\_MITOCHONDRIAL\_MATRIX**

  
