# Supplementary material for: A novel signature model based on mitochondrial-related genes for predicting survival of colon adenocarcinoma
Source: BMC Med Inform Decis Mak. 2022 Oct 22;22:277. doi: 10.1186/s12911-022-02020-3 (PMC9587559; doi:10.1186/s12911-022-02020-3)
Supplement: Supplementary file 2 — Additional file 2. Raw data. (ZIP 320499 kb) [file 12911_2022_2020_MOESM2_ESM.zip › Raw data/5. GSEA Result/GSEA_RESULT/GOBP_REGULATION_OF_MITOCHONDRIAL_GENE_EXPRESSION.html]

Details for gene set GOBP\_REGULATION\_OF\_MITOCHONDRIAL\_GENE\_EXPRESSION[GSEA]

|  || Dataset | input.input.cls#T\_versus\_N.input.cls#T\_versus\_N\_repos |
| Phenotype | input.cls#T\_versus\_N\_repos |
| Upregulated in class | T |
| GeneSet | GOBP\_REGULATION\_OF\_MITOCHONDRIAL\_GENE\_EXPRESSION |
| Enrichment Score (ES) | 0.82887053 |
| Normalized Enrichment Score (NES) | 1.9864631 |
| Nominal p-value | 0.0 |
| FDR q-value | 0.0060096155 |
| FWER p-Value | 0.004 |
Table: GSEA Results Summary

  

Fig 1: Enrichment plot: GOBP\_REGULATION\_OF\_MITOCHONDRIAL\_GENE\_EXPRESSION      
 Profile of the Running ES Score & Positions of GeneSet Members on the Rank Ordered List

  

| SYMBOL | TITLE | RANK IN GENE LIST | RANK METRIC SCORE | RUNNING ES | CORE ENRICHMENT || 1 | RPUSD4 | na | 124 | 1.239 | 0.0745 | Yes |
| 2 | CDK5RAP1 | na | 234 | 1.157 | 0.1441 | Yes |
| 3 | MALSU1 | na | 397 | 1.076 | 0.2079 | Yes |
| 4 | SHMT2 | na | 434 | 1.061 | 0.2729 | Yes |
| 5 | MTG2 | na | 709 | 0.963 | 0.3276 | Yes |
| 6 | RPUSD3 | na | 1215 | 0.848 | 0.3710 | Yes |
| 7 | FASTKD2 | na | 1713 | 0.764 | 0.4093 | Yes |
| 8 | UQCC1 | na | 1940 | 0.732 | 0.4505 | Yes |
| 9 | MRPS27 | na | 2449 | 0.671 | 0.4829 | Yes |
| 10 | TRMT10C | na | 2763 | 0.633 | 0.5165 | Yes |
| 11 | UQCC2 | na | 2802 | 0.630 | 0.5548 | Yes |
| 12 | MTG1 | na | 3077 | 0.604 | 0.5872 | Yes |
| 13 | RCC1L | na | 3242 | 0.590 | 0.6208 | Yes |
| 14 | TRUB2 | na | 3257 | 0.589 | 0.6570 | Yes |
| 15 | RMND1 | na | 3283 | 0.586 | 0.6928 | Yes |
| 16 | TSFM | na | 3381 | 0.579 | 0.7269 | Yes |
| 17 | LRPPRC | na | 3389 | 0.579 | 0.7627 | Yes |
| 18 | MPV17L2 | na | 3579 | 0.563 | 0.7941 | Yes |
| 19 | C1QBP | na | 3584 | 0.562 | 0.8289 | Yes |
| 20 | ALKBH1 | na | 12618 | 0.224 | 0.6792 | No |
| 21 | FASTKD3 | na | 13305 | 0.211 | 0.6798 | No |
| 22 | NGRN | na | 14792 | 0.189 | 0.6646 | No |
| 23 | METTL4 | na | 36539 | 0.019 | 0.2721 | No |
| 24 | PRKAA1 | na | 45733 | -0.064 | 0.1097 | No |
| 25 | COA3 | na | 45972 | -0.072 | 0.1098 | No |
| 26 | NSUN3 | na | 48104 | -0.156 | 0.0809 | No |
| 27 | TACO1 | na | 50275 | -0.283 | 0.0591 | No |
| 28 | CHCHD10 | na | 52698 | -0.505 | 0.0465 | No |
Table: GSEA details [plain text format]

  

Fig 2: GOBP\_REGULATION\_OF\_MITOCHONDRIAL\_GENE\_EXPRESSION      
 Blue-Pink O' Gram in the Space of the Analyzed GeneSet

  

Fig 3: GOBP\_REGULATION\_OF\_MITOCHONDRIAL\_GENE\_EXPRESSION: Random ES distribution      
 Gene set null distribution of ES for **GOBP\_REGULATION\_OF\_MITOCHONDRIAL\_GENE\_EXPRESSION**

  
