# Supplementary material for: A novel signature model based on mitochondrial-related genes for predicting survival of colon adenocarcinoma
Source: BMC Med Inform Decis Mak. 2022 Oct 22;22:277. doi: 10.1186/s12911-022-02020-3 (PMC9587559; doi:10.1186/s12911-022-02020-3)
Supplement: Supplementary file 2 — Additional file 2. Raw data. (ZIP 320499 kb) [file 12911_2022_2020_MOESM2_ESM.zip › Raw data/5. GSEA Result/GSEA_RESULT/gsea_report_for_N_1618446927360.html]

Report for N 1618446927360 [GSEA]

| GS  follow link to MSigDB | GS DETAILS | SIZE | ES | NES | NOM p-val | FDR q-val | FWER p-val | RANK AT MAX | LEADING EDGE |Table: Gene sets enriched in phenotype **N (41 samples)**[plain text format]****

  
