# Supplementary material for: A novel signature model based on mitochondrial-related genes for predicting survival of colon adenocarcinoma
Source: BMC Med Inform Decis Mak. 2022 Oct 22;22:277. doi: 10.1186/s12911-022-02020-3 (PMC9587559; doi:10.1186/s12911-022-02020-3)
Supplement: Supplementary file 2 — Additional file 2. Raw data. (ZIP 320499 kb) [file 12911_2022_2020_MOESM2_ESM.zip › Raw data/5. GSEA Result/GSEA_RESULT/gsea_report_for_T_1618446927360.html]

Report for T 1618446927360 [GSEA]

| GS  follow link to MSigDB | GS DETAILS | SIZE | ES | NES | NOM p-val | FDR q-val | FWER p-val | RANK AT MAX | LEADING EDGE || 1 | GOBP\_REGULATION\_OF\_MITOCHONDRIAL\_GENE\_EXPRESSION | Details ... | 28 | 0.83 | 1.99 | 0.000 | 0.006 | 0.004 | 3584 | tags=68%, list=6%, signal=73% |
| 2 | GOBP\_MITOCHONDRIAL\_RNA\_METABOLIC\_PROCESS | Details ... | 44 | 0.71 | 1.87 | 0.012 | 0.008 | 0.010 | 10154 | tags=75%, list=18%, signal=92% |
| 3 | GOBP\_POSITIVE\_REGULATION\_OF\_MITOCHONDRIAL\_TRANSLATION | Details ... | 16 | 0.89 | 1.87 | 0.000 | 0.006 | 0.010 | 3584 | tags=81%, list=6%, signal=87% |
| 4 | GOBP\_MITOCHONDRIAL\_RNA\_PROCESSING | Details ... | 16 | 0.83 | 1.87 | 0.000 | 0.005 | 0.011 | 5467 | tags=81%, list=10%, signal=90% |
| 5 | GOBP\_MITOCHONDRIAL\_GENE\_EXPRESSION | Details ... | 161 | 0.71 | 1.77 | 0.030 | 0.018 | 0.035 | 7928 | tags=65%, list=14%, signal=76% |
| 6 | GOBP\_PROTEIN\_IMPORT\_INTO\_MITOCHONDRIAL\_MATRIX | Details ... | 20 | 0.73 | 1.75 | 0.012 | 0.019 | 0.041 | 8594 | tags=80%, list=16%, signal=95% |
| 7 | GOBP\_MITOCHONDRIAL\_TRANSLATION | Details ... | 132 | 0.72 | 1.73 | 0.041 | 0.020 | 0.043 | 7928 | tags=67%, list=14%, signal=79% |
| 8 | GOBP\_MITOCHONDRIAL\_GENOME\_MAINTENANCE | Details ... | 21 | 0.58 | 1.68 | 0.015 | 0.025 | 0.064 | 3623 | tags=48%, list=7%, signal=51% |
| 9 | GOBP\_POSITIVE\_REGULATION\_OF\_MITOCHONDRIAL\_OUTER\_MEMBRANE\_PERMEABILIZATION\_INVOLVED\_IN\_APOPTOTIC\_SIGNALING\_PATHWAY | Details ... | 35 | 0.55 | 1.61 | 0.028 | 0.037 | 0.092 | 9343 | tags=63%, list=17%, signal=76% |
Table: Gene sets enriched in phenotype **T (480 samples)**[plain text format]****

  
