# Supplementary material for: A novel signature model based on mitochondrial-related genes for predicting survival of colon adenocarcinoma
Source: BMC Med Inform Decis Mak. 2022 Oct 22;22:277. doi: 10.1186/s12911-022-02020-3 (PMC9587559; doi:10.1186/s12911-022-02020-3)
Supplement: Supplementary file 2 — Additional file 2. Raw data. (ZIP 320499 kb) [file 12911_2022_2020_MOESM2_ESM.zip › Raw data/5. GSEA Result/GSEA_RESULT/heat_map_corr_plot.html]

Heat map and correlation plot for input.input.cls#T\_versus\_N  

Fig 1: heat\_map      
 Heat Map of the top 50 features for each phenotype in input.input.cls#T\_versus\_N

  
  

Fig 2: Ranked Gene List Correlation Profile      
 Ranked list correlations for input.input.cls#T\_versus\_N

  
  
    
