# Supplementary material for: A novel signature model based on mitochondrial-related genes for predicting survival of colon adenocarcinoma
Source: BMC Med Inform Decis Mak. 2022 Oct 22;22:277. doi: 10.1186/s12911-022-02020-3 (PMC9587559; doi:10.1186/s12911-022-02020-3)
Supplement: Supplementary file 2 — Additional file 2. Raw data. (ZIP 320499 kb) [file 12911_2022_2020_MOESM2_ESM.zip › Raw data/5. GSEA Result/GSEA_RESULT/index.html]

Index for xtools.gsea.Gsea COADNEW.Gsea.1618446927360

### GSEA Report for Dataset input

#### Enrichment in phenotype: **T (480 samples)**

- 9 / 9 gene sets are upregulated in phenotype **T**- 9 gene sets are significant at FDR < 25%- 3 gene sets are significantly enriched at nominal pvalue < 1%- 9 gene sets are significantly enriched at nominal pvalue < 5%- Snapshot of enrichment results- Detailed enrichment results in html format- Detailed enrichment results in TSV format (tab delimited text)- Guide to interpret results

#### Enrichment in phenotype: **N (41 samples)**

- None of the gene sets are enriched in phenotype **N**- Guide to interpret results

#### Dataset details

- The dataset has 55268 features (genes)- No probe set => gene symbol collapsing was requested, so all 55268 features were used

#### Gene set details

- Gene set size filters (min=15, max=500) resulted in filtering out 0 / 9 gene sets- The remaining 9 gene sets were used in the analysis- List of gene sets used and their sizes (restricted to features in the specified dataset)

#### Gene markers for the **T** *versus* **N** comparison

- The dataset has 55268 features (genes)- # of markers for phenotype **T**: 41550 (75.2% ) with correlation area 69.5%- # of markers for phenotype **N**: 13718 (24.8% ) with correlation area 30.5%- Detailed rank ordered gene list for all features in the dataset- Heat map and gene list correlation  profile for all features in the dataset- Butterfly plot of significant genes

#### Global statistics and plots

- Plot of p-values *vs.* NES- Global ES histogram

#### Other

- Parameters used for this analysis

#### Comments

- There were duplicate row identifiers in the specified dataset. One id was arbitarilly choosen. Details are below
  Generally, this is OK, but if you want to avoid this automagic, edit your dataset so that all row ids are unique
  # of row ids in original dataset: 56753
  # of row UNIQUE ids in original dataset: 55268
  # The duplicates were
  RF00019
  RF00019
  RF00019
  RF00019
  RF00019
  RF00019
  RF00019
  RF00019
  RF00017
  RF00019
  RF00019
  RF00019
  RF00019
  RF00019
  RF00017
  RF00019
  RF00017
  RF00019
  RF00017
  RF00019
  RF00019
  RF00019
  RF00019
  RF00017
  RF00019
  RF00017
  RF00019
  RF00019
  RF00019
  RF00019
  RF00019
  RF00017
  RF00019
  RF00019
  RF00019
  RF00019
  RF00019
  RF00019
  RF00019
  RF00019
  RF00019
  RF00156
  RF00017
  RF00019
  RF00017
  RF00019
  RF00156
  RF00019
  RF00019
  RF00019
  RF00017
  RF00012
  RF00017
  RF00019
  RF00019
  RF00019
  RF00019
  RF00003
  RF00019
  RF00017
  RF00004
  RF00017
  RF00017
  RF00156
  RF00019
  RF00019
  RAET1E-AS1
  RF00019
  RF00019
  RF00019
  RF00156
  RF00019
  RF00019
  RF01210
  RF00019
  RF00017
  RF00019
  RF00019
  RF00019
  RF00017
  RF00019
  RF00019
  RF00019
  RF00019
  RF00019
  RF00017
  RF00012
  RF01210
  RF00019
  RF00019
  RF00019
  RF00017
  RF00019
  RF00322
  RF00019
  RF00322
  RF00019
  RF00017
  RF01210
  RF00017
  RF00019
  RF00019
  RF00019
  RF00017
  RF00017
  RF02110
  RF00019
  RF00019
  RF00017
  RF00156
  RF01210
  RF00019
  RF00019
  RF00003
  RF00017
  RF00012
  RF00019
  RF00019
  RF00019
  RF00019
  RF00019
  RF00019
  RF00019
  RF00017
  RF00017
  RF00432
  RF02271
  RF00019
  RF01210
  RF00019
  RF00019
  RF00012
  RF00019
  RF00019
  RF00019
  RF00019
  RF00019
  RF00402
  RF00432
  RF00408
  RF00019
  RF00402
  RF00019
  RF00019
  RF00017
  RF00012
  RF02110
  RF00019
  RF00019
  RF00019
  RF00017
  RF00096
  RF00019
  RF00019
  RF00019
  RF00019
  RF01518
  RF00019
  RF00017
  RF00426
  RF00019
  RF00092
  RF00019
  RF00019
  RF00019
  RF00019
  RF00019
  RF00019
  RF00019
  RF00019
  RF00322
  RF00402
  RF00017
  RF00402
  RF00019
  RF00019
  RF00019
  RF00019
  RF00432
  RF00156
  RF00019
  RF01225
  RF00019
  RF00019
  RF00019
  RF02271
  RF00019
  RF02271
  RF00432
  RF00017
  RF02271
  RF00019
  RF00017
  RF00012
  RF00019
  RF00019
  RF00019
  RF00017
  RF00019
  RF00019
  RF00019
  RF00019
  RF00019
  RF00017
  LINC01422
  RF00017
  RF00019
  RF00019
  RF00019
  RF00019
  RF00019
  RF00561
  RF00019
  RF00019
  RF00019
  RF00017
  RF00019
  RF00416
  RF00591
  RF00019
  RF00019
  RF00019
  RF00019
  RF00019
  RF00019
  RF00019
  RF00554
  RF00019
  RF00019
  RF00019
  RF00017
  RF00017
  RF00019
  RF00019
  RF00019
  RF00003
  RF00017
  RF00272
  RF00096
  RF00019
  RF00017
  RF00019
  RF00283
  RF00019
  RF00019
  RF00017
  RF00019
  RF00438
  RF00019
  RF00004
  RF00019
  RF00019
  RF00019
  RF00019
  RF00561
  RF00019
  RF00019
  RF01210
  RF00019
  RF00019
  RF00019
  RF00012
  RF00019
  RF00272
  RF00012
  RF00019
  RF00019
  RF00012
  RF01169
  RF00019
  RF00017
  RF00019
  RF00019
  RF01210
  RF00019
  RF00012
  RF00139
  RF00019
  RF00019
  RF00019
  RF00402
  RF00181
  RF00019
  RF00019
  RF02271
  RF00561
  RF00019
  RF00019
  RF00019
  RF00017
  RF00019
  RF00139
  RF00019
  RF00019
  RF00019
  RF00017
  RF00554
  RF00019
  RF00019
  RF00019
  RF00019
  RF00019
  RF00019
  RF00017
  RF00402
  RF00019
  RF00019
  RF00019
  RF00019
  RF00017
  RF01210
  RF00017
  RF00019
  RF00019
  RF00019
  RF00019
  RF00019
  RF00019
  RF00019
  RF00019
  RF00322
  RF00019
  RF00019
  RF00019
  RF00019
  RF00017
  RF00004
  RF00017
  RF00026
  RF00019
  RF00019
  RF00019
  RF00096
  RF00017
  RF00019
  RF00019
  RF00096
  RF02271
  RF00019
  RF00019
  RF00019
  RF00019
  RF00019
  BMS1P4
  RF00066
  RF00393
  RF00019
  RF00019
  RF00019
  RF00019
  RF00096
  RF00601
  RF02091
  RF00019
  RF00019
  RF00019
  RF00026
  RF02271
  RF00017
  RF00017
  RF00019
  RF00322
  RF00019
  RF00019
  RF00322
  RF00004
  RF00019
  RF00019
  RF00019
  RF00322
  RF00019
  RF00019
  RF00019
  RF00019
  RF00019
  RF00322
  RF00012
  RF00019
  RF00019
  RF00012
  RF00019
  RF00019
  RF00019
  RF00019
  RF00019
  RF00019
  RF00139
  RF00561
  RF00019
  RF00019
  RF00019
  RF01293
  RF00264
  RF00402
  RF00072
  RF00264
  RF00019
  LINC01505
  RF00019
  RF00019
  DNAJC9-AS1
  RF00156
  RF00019
  RF00096
  RF00019
  RF00019
  RF00012
  RF00019
  LINC01115
  RF00017
  RF00272
  RF00019
  RF00091
  RF00154
  RF00019
  RF00019
  SNHG28
  RF00017
  RF00019
  RF00019
  RF00019
  RF00402
  RF00012
  RF00019
  RF00019
  RF00012
  RF00561
  RF00019
  RF00019
  RF00612
  RF00413
  RF00019
  RF00019
  RF00017
  RF00019
  RF02271
  RF00017
  RF00017
  RF00019
  RF00066
  RF00096
  SNORA16A
  RF00100
  RF00019
  RF00402
  RF00393
  RF00019
  RF00191
  RF00019
  RF00322
  RF00019
  RF00284
  RF00012
  RF00019
  RF00263
  RF00017
  RF00019
  RF00019
  RF00019
  RF00019
  RF01210
  RF00019
  RF00019
  RF00019
  RF00019
  RF00019
  RF00019
  RF02271
  RF00402
  RF00019
  RF00019
  RF00019
  RF00019
  RF00019
  RF00019
  RF00019
  RF00012
  RF00019
  RF00019
  RF00019
  RF00019
  RF00019
  RF00019
  RF00322
  RF00019
  RF00443
  RF00017
  RF00006
  RF00561
  RF00181
  RF00019
  RF00019
  RF00592
  RF00019
  RF00568
  RF00019
  RF00019
  SPATA13
  RF00002
  RF00322
  RF00019
  RF00019
  RF00019
  RF00019
  RF01210
  RF00019
  RF02271
  RF00568
  RF00561
  RF00019
  RF00019
  RF01210
  RF00003
  RF00019
  RF00019
  RF00017
  RF00019
  RF00019
  RF00019
  RF00017
  RF02271
  RF00322
  RF00019
  RF00416
  RF00017
  RF00019
  RF00019
  RF00019
  RF00019
  RF00012
  RF00003
  RF00564
  RF00019
  RF00017
  RF00019
  RF00019
  RF00019
  RF00019
  RF00019
  RF00019
  RF00602
  RF00334
  RF02271
  RF00090
  RF00026
  RF00019
  RF00004
  RF00019
  RF00012
  RF00019
  RF00019
  RF00019
  RF00019
  RF01518
  RF00019
  RF00017
  RF00422
  RF00019
  RF00019
  RF01210
  RF00017
  RF00568
  RF00091
  RF00411
  RF00401
  RF00019
  RF00275
  RF00443
  RF00157
  RF00017
  RF00334
  RF00004
  RF00264
  RF00019
  RF00601
  RF00092
  RF00017
  RF00019
  RF00019
  RF00019
  RF00017
  RF00019
  RF00012
  RF00019
  RF01518
  RF00019
  RF00432
  RF00272
  RF00554
  RF00019
  RF00017
  RF00019
  RF00019
  RF00019
  RF00017
  RF00019
  RF00019
  RF00402
  RF00019
  RF00017
  RF00004
  RF00019
  RF00019
  RF00019
  RF00019
  RF00017
  RF00019
  RF00614
  RF01210
  RF00322
  RF00019
  RF00019
  RF00019
  RF00072
  RF00409
  RF00019
  RF00279
  RF01210
  RF00019
  RF00019
  RF00012
  RF00019
  RF00019
  RF00402
  RF00019
  RF00402
  RF00017
  RF00019
  RF00019
  RF01210
  RF00019
  RF00599
  RF00560
  RF00019
  RF00019
  RF00019
  RF00019
  HOXC5
  RF00017
  RF00004
  RF00019
  RF00019
  RF00561
  RF00017
  RF00019
  RF00019
  RF00019
  RF00019
  RF00554
  RF00438
  RF00156
  RF00568
  RF00019
  RF00019
  RF00019
  RF00019
  RF00019
  RF00019
  RF00019
  RF00019
  RF00019
  RF00017
  RF00012
  RF00019
  RF00019
  RF00334
  RF00019
  RF00017
  RF00019
  RF00019
  RF00181
  RF00139
  RF00045
  RF00019
  RF00212
  RF00156
  RF01241
  RF00015
  RF00096
  RF00561
  RF00019
  RF00212
  RF00019
  RF00096
  RF00019
  RF00019
  RF00019
  RF00019
  RF00019
  RF00019
  RF00100
  RF01684
  RF00601
  RF00156
  RF00019
  RF00409
  RF02108
  RF00019
  RF01233
  RF02107
  RF00019
  RF00019
  ALG1L9P
  RF00019
  RF01210
  RF00275
  RF00019
  RF02171
  RF00019
  RF00560
  RF01518
  RF00092
  RF00072
  RF00019
  RF00017
  RF00019
  RF00561
  RF02271
  RF01210
  RF02271
  RF00019
  RF00019
  RF00096
  RF00019
  RF00012
  RF00019
  RF00019
  RF00284
  RF00019
  RF00019
  RF01210
  GOLGA8M
  RF00019
  RF00019
  RF00017
  RF00019
  RF00019
  RF00019
  RF00019
  RF02271
  RF00019
  RF00019
  RF00019
  RF00191
  RF02105
  RF00012
  RF00019
  RF00019
  RF00591
  RF02271
  RF00019
  RF00019
  RF00019
  RF00019
  RF00017
  RF00019
  RF00019
  RF00284
  RF00019
  RF00554
  RF00019
  RF00019
  RF00019
  RF00019
  RF00275
  RF00019
  RF00100
  RF00019
  RF00017
  RF00284
  RF00019
  RF00056
  RF00019
  RF00017
  RF00284
  RF02271
  RF00092
  RF00019
  RF00019
  RF00019
  RF00019
  RF00019
  RF00019
  RF00561
  RF00003
  RF02271
  RF00017
  RF00019
  RF00136
  RF00019
  RF00139
  RF00019
  RF01225
  RF00568
  RF00096
  RF00322
  RF00004
  RF00012
  POLR2J4
  RF00026
  RF00019
  RF00019
  RF00150
  RF00026
  RF00568
  RF00614
  RF00017
  RF00019
  RF00017
  RF01233
  RF00019
  RF00017
  RF00019
  RF02271
  SNX29P2
  RF00019
  RF00012
  RF00019
  RF00019
  RF00019
  RF00017
  RF01233
  RF00003
  RF02271
  RF00564
  RF00019
  RF00017
  GUSBP9
  RF00019
  RF00012
  RF00019
  RF00019
  RNA5-8SN2
  RF00019
  RF00019
  RF00416
  RF00322
  RF00019
  RF00093
  RF00019
  RF00019
  RF00019
  RF00003
  RF00402
  RF00285
  RGS5
  RF00019
  RF00410
  RF00012
  RF00017
  RF02271
  RF00019
  RF00561
  RF00492
  RF00019
  RF00096
  RF00273
  ZNF503
  RF02116
  RF00019
  RF00019
  RF00019
  RF00019
  RF00019
  RF02271
  RF00017
  RF02271
  RF00100
  RF00017
  RF00019
  RF00019
  RF00017
  RF00019
  RF00408
  RF00156
  RF00017
  RF00019
  RF00019
  RF00156
  RF00019
  RF00019
  RF00561
  RF01210
  RF00019
  RF00019
  RF00017
  RF00019
  RF00019
  RF02271
  RF00019
  RF00019
  RF00019
  RF01210
  RF00012
  RF00096
  RF00019
  RF00019
  RF00015
  RF00090
  RF00322
  RF00019
  RF00019
  RF00096
  RF00004
  RF00019
  RF00017
  RF00091
  RF01210
  RF00100
  RF00019
  RF00279
  RF00554
  RF00425
  RF00004
  RF00019
  RF00019
  RF00019
  RF00019
  RF00322
  RF00017
  RF00019
  RF00019
  RF00019
  RF00568
  RF00019
  RF00019
  RF00045
  RF00004
  RF00019
  RF00017
  RF00019
  RF00019
  RF00017
  RF00019
  RF00019
  RF01241
  RF00017
  RF00019
  RF00017
  RF00019
  RF00279
  RF00017
  RF01225
  RF00019
  RF00425
  RF00019
  RF01518
  RF02271
  RF00019
  RF01210
  LINC00484
  RF00019
  RF00561
  RF00554
  RF00019
  RF00619
  RF00017
  RF00019
  RF00019
  RF00019
  RF00019
  RF00019
  RF00019
  RF00156
  RF00568
  RF00561
  RF00019
  RF00443
  RF00019
  RF00017
  RF00019
  RF00432
  RF00271
  RF00066
  RF00019
  RF00006
  RF00019
  SNORD3D
  RF00139
  RF00019
  RF00019
  RF00019
  RF00017
  RF00019
  RF00019
  RF00156
  RF00019
  RF00432
  RF00019
  RF00019
  RF00019
  RF00019
  RF00019
  RF00019
  RF00017
  RF00019
  RF00017
  RF00019
  DUXAP8
  RF00017
  RF00019
  RF00156
  RF00494
  RF00019
  RF00017
  RF00019
  RF00019
  RF01518
  RF00012
  RF00599
  RF00601
  RF00019
  RF00012
  RF00019
  RF02109
  RF00004
  RF00019
  RF00019
  RF02271
  RF00019
  RF00019
  RF00019
  RF00019
  RF00017
  RF00017
  PRICKLE2-AS1
  RF00019
  RF00019
  RF00019
  RF00440
  RF00019
  RF00156
  RF00017
  RF00402
  CYB561D2
  RF00019
  RF00017
  RF00072
  RF02106
  RF00019
  RF00019
  RF00019
  RF00015
  RF00019
  RF00012
  RF00432
  RF00019
  RF00019
  RF00019
  RF00019
  H2BFS
  RF00019
  RF00019
  RF00019
  RF00019
  RF00019
  RF00019
  RF00019
  RF01210
  RF00413
  RF00003
  RF00019
  RF00554
  RF00019
  RF00019
  RF00019
  RF00017
  RF00017
  RF00017
  RF00012
  RF00017
  RF00096
  RF00019
  RF00019
  RF00056
  RF00019
  RF00017
  RF00019
  RF00019
  RF00019
  RF00017
  RF00019
  RF00019
  RF00322
  RF00012
  RF00019
  RF00019
  RF00322
  RF00019
  RF00019
  RF00019
  RF00409
  RF00561
  RF00066
  RF00019
  RF00019
  RF00001
  RF00019
  RF00561
  RF00019
  RF00019
  RF00561
  RF00568
  SCARNA4
  RF00156
  RF00096
  RF00019
  RF00100
  RF00019
  RF00494
  RF00601
  RF00001
  RF00019
  RF00019
  RF00019
  RF00019
  RF00019
  RF00156
  RF00019
  RF00019
  RF00154
  RF00019
  RF00425
  RF00019
  RF00019
  RF01233
  RF00394
  RF00017
  RF00019
  RF00019
  RF00017
  RF00019
  RF01210
  RF00019
  RF00275
  RF00017
  RF00156
  RF00019
  RF00019
  RF00019
  RF00012
  RF00019
  RF00272
  RF00017
  RF00108
  RF00402
  RF00263
  RF00569
  RF00019
  RF00019
  RF00019
  RF02271
  RF00012
  RF00156
  RF00012
  RF00324
  LINC01481
  RF00019
  RF00019
  RF00425
  SNORA17B
  RF00019
  RF00017
  RF00017
  RF00072
  RF02271
  RF00425
  RF00019
  RF00096
  RF00017
  RF00598
  RF00017
  RF00425
  RF00019
  RF00017
  RF00012
  RF00019
  RF00410
  RF00019
  RF00012
  RF00019
  RF00019
  RF00019
  RF00156
  RF00012
  RF00019
  RF00019
  RF00019
  RF00190
  RF00017
  RF00004
  RF00019
  RF00019
  RF00019
  RF00019
  RF00019
  RF01161
  RF00019
  RF00019
  RF00017
  RMRP
  RF00322
  RF00019
  LINC01238
  RF00003
  RF00019
  RF00560
  RF00017
  RF00421
  RF00026
  RF00019
  RF00019
  RNA5-8SN2
  RF00019
  RF00017
  RF00019
  RF00019
  RF00017
  RF00019
  RF00156
  RF00322
  RF00004
  RF00092
  RF00015
  RF00019
  RF00019
  RF00492
  RF00409
  RF00394
  RF00019
  RF00019
  RF00402
  RF00424
  RF00019
  RF00322
  RF00017
  RF00401
  RF00017
  RF00019
  RF00019
  RF00019
  RF00017
  RF00092
  RF00019
  RF00019
  RF00019
  RF00432
  RF00394
  MATR3
  SNORA50D
  RF00019
  RNA5-8SN2
  RF00019
  RF00019
  RF00017
  RF00017
  RF00019
  RF00019
  RF00156
  RF00019
  RF00136
  RF00406
  RF00019
  RF00019
  RF00019
  RF00019
  RF02271
  RF00017
  RF00019
  RF00424
  RF00026
  RF00019
  RF00019
  RF00019
  RF00019
  RF00091
  RF00019
  RF00285
  RF00019
  RF00003
  RF00019
  RF00571
  RF00019
  LINC-PINT
  RF00019
  RF00017
  RF00108
  RF00278
  RF00432
  RF01210
  RF00411
  RF00421
  RF00019
  RF00019
  RF00492
  RF00494
  RF00019
  RF00090
  RF00012
  RF00012
  ELFN2
  RF00019
  RF00019
  RF00019
  RF00614
  RF00017
  RF00019
  RF00019
  RF00019
  RF00019
  RF00019
  RF01210
  RF00157
  RF00019
  RF00017
  RF00017
  SNORA50A
  RF01518
  RF00322
  RF00416
  RF00019
  RF00019
  RF00015
  RF00019
  RF01225
  RF01233
  RF00017
  RF00424
  RF00096
  RF00393
  RF00012
  RF00017
  RF00156
  RF02271
  RF00003
  RF00017
  RF00019
  RF00554
  RF00614
  RF00017
  RF00019
  RF00181
  RF00019
  RF00432
  RF01225
  RF00019
  RF00157
  RF00019
  RF01210
  RF00012
  RF00493
  RF00019
  RF00019
  RF00019
  RF00019
  RF00019
  RF00019
  RF00019
  RF00017
  RF00091
  RF00019
  RF00019
  PKD1P1
  RF00019
  RF00285
  RF00560
  RF00066
  RF00006
  RF00019
  RF00012
  RF00012
  RF00017
  RF00019
  RF02271
  ITFG2-AS1
  RF00012
  RF00019
  RF00045
  RF00019
  RF00096
  RF00019
  RF00017
  RF00019
  RF00019
  RF00017
  RF00017
  RF02271
  RF00019
  RF00212
  RF00017
  RF00017
  RF00019
  RF00156
  RF02271
  RF00017
  RF00105
  RF00017
  SNORD38B
  RF00019
  RF02219
  RF00019
  RF00019
  RF00019
  RF00019
  RF00322
  RF00019
  TMSB15B
  RF00272
  RF00340
  RF00019
  RF00019
  RF00275
  RF00019
  RF00019
  RF00017
  RF00019
  RF00019
  RF00019
  RF00019
  RF00019
  RF00017
  RF00017
  RF00614
  RF00019
  RF00019
  RF00019
  RF00554
  RF00017
  RF00019
  RF00019
  SNORD3E
  RF01210
  RF00019
  RF00092
  RF00019
  RF00019
  RF00017
  RF00096
  SNORA50B
  RF00019
  RF00026
  RF00004
  RF00019
  RF02271
  LINC01297
  RF00181
  RF00019
  RF00096
  RF00322
  RF00591
  RF00017
  CTSLP2
  RF00019
  RF02271
  RF00017
  RF01210
  RF00012
  RF00279
  RF00019
  RF00019
  RF00019
  RF01210
  RF00019
  PINX1
  RF00019
  RF00586
  RF00012
  RF00218
  RF00019
  RF00019
  RF00017
  RF00019
  RF00424
  RF00263
  RF00019
  RF00426
  RF00066
  RF00019
  RF00156
  RF00406
  RF00012
  RF00017
  RF00019
  RF00017
  RF01518- Timestamp used as random seed: 1618446927859

---

Report: COADNEW.Gsea.1618446927360.rpt   by user: TIAN

xtools.gsea.Gsea [Thu, Apr 15, '21 8 AM 35]

Website: www.gsea-msigdb.org/gsea
Questions & Suggestions: Contact page
