# Supplementary material for: A novel signature model based on mitochondrial-related genes for predicting survival of colon adenocarcinoma
Source: BMC Med Inform Decis Mak. 2022 Oct 22;22:277. doi: 10.1186/s12911-022-02020-3 (PMC9587559; doi:10.1186/s12911-022-02020-3)
Supplement: Supplementary file 2 — Additional file 2. Raw data. (ZIP 320499 kb) [file 12911_2022_2020_MOESM2_ESM.zip › Raw data/5. GSEA Result/GSEA_RESULT/pos_snapshot.html]

Snapshot of 9 enrichment plots

|  |  |  |
| --- | --- | --- |
|  |  |  |
|  |  |  |
|  |  |  |
Table: Snapshot of enrichment results

  
